# Paeoniflorin protects against NAFLD through antioxidant, anti-inflammatory effects and restoration of gut microbiota homeostasis

**DOI:** 10.3389/fmicb.2026.1766068

**Published:** 2026-03-13

**Authors:** Xiao-Long Wang, Chang Zhang, De-Shuang Lu, Zhuo-Yue Dong, Bai-Ming Jin, Si-Yuan Wan, Zi-Wen Zhang, Chun-Jing Zhang, Lin Li

**Affiliations:** 1 School of Biomedical Engineering, Guangzhou Medical University, Guangzhou, Guangdong, China; 2 Department of Medical Technology, Qiqihar Medical University, Qiqihar, Heilongjiang, China; 3 College of Public Health, Qiqihar Medical University, Qiqihar, Heilongjiang, China; 4 Heilongjiang Provincial Key Laboratory of Food and Medicine Homology and Metabolic Disease Prevention, Qiqihar Medical University, Qiqihar, Heilongjiang, China

**Keywords:** glycolipid metabolism, gut microbiota, intestinal barrier, NAFLD, oxidative stress, paeoniflorin

## Abstract

Non-alcoholic fatty liver disease (NAFLD) is a widespread chronic metabolic disorder characterized by hepatic lipid accumulation, oxidative stress, inflammation and gut dysbiosis. Paeoniflorin (PAF) exhibits potential against NAFLD, yet its antioxidant mechanism via the gut-liver axis remains unclear. In a high-fat/sucrose (HFS) diet-induced NAFLD mouse model, C57BL/6 mice received PAF (50 or 100 mg/kg/day) for 10 weeks. Oxidative stress markers, histopathology, gut microbiota, and serum metabolomics were conducted, with fecal microbiota transplantation (FMT) applied for causal validation. PAF ameliorated metabolic disorders by suppressing hepatic lipogenesis and promoting cholesterol excretion. PAF significantly ameliorated oxidative stress by enhancing hepatic and colonic anti-oxidant capacity, evidenced by increased SOD activity and decreased MDA levels. It concurrently reduced systemic inflammation and enhanced intestinal barrier integrity via upregulation of tight junction proteins. Furthermore, PAF reshaped the gut microbiota, elevating beneficial *Akkermansia* and microbial-derived SCFAs, while suppressing pro-oxidant and pro-inflammatory pathogens like Desulfovibrio and Helicobacter. FMT confirmed that these antioxidant and metabolic benefits were mediated by the gut microbiota. In conclusion, PAF alleviates NAFLD primarily through potent antioxidant actions and anti-inflammatory, achieved via remodeling gut microbial ecology and reinforcing intestinal barrier.

## 1 Introduction

With the continuous rise in global obesity rates, obesity-induced non-alcoholic fatty liver disease (NAFLD) has emerged as a critical public health concern (Song et al., 2025). NAFLD not only leads to hepatic dysfunction but also closely associated with various metabolic disorders, including cardiovascular diseases and type 2 diabetes, significantly compromising patients’ quality of life and life expectancy (Ji et al., 2024; Ni et al., 2024). Overeating disrupts the balance of glucose and lipid metabolism, primarily by overwhelming the regulatory capacity for energy surplus in the liver and adipose tissue (Piché et al., 2020). In NAFLD, insulin resistance (IR) initiates a cascade of metabolic dysfunction. IR directly causes hyperinsulinemia, which stimulates adipose tissue lipolysis. The resulting influx of free fatty acids to the liver drives hepatic steatosis and disrupts both glucose and lipid metabolism (Muzurović et al., 2021). The accumulation of lipids is driven by a metabolic imbalance between lipid acquisition (through diet and *de novo* lipogenesis) and lipid clearance (via β-oxidation and export) (Guo et al., 2022). Lipid-induced ROS overproduction depletes antioxidant capacity such as SOD, GPx, leading to oxidative stress, evidenced by markers like MDA, that impairs hepatocyte and mitochondrial function, thereby worsening metabolic and inflammatory state (Rives et al., 2020; Notarnicola et al., 2021; Chen et al., 2023). The persistent injury triggered by lipid accumulation and oxidative stress induce Kupffer cell polarization toward a pro-inflammatory M1 phenotype, which secretes cytokines such as TNF-α, IL-1β, IL-6, leading to the hepatic and systemic inflammation that thereby exacerbate hepatocyte injury (Barreby et al., 2022). Given that the liver is anatomically connected to the gut via the portal vein, it is particularly susceptible to bacterial translocation (Tripathi et al., 2018). Consequently, bacteria, bacterial metabolites, lipopolysaccharides (LPS), and inflammatory cytokines can migrate to the liver (Shao et al., 2024). Accumulating evidence indicates that gut microbiota dysbiosis is closely linked to the pathogenesis of NAFLD (Fan et al., 2023). Comparative studies involving healthy individuals, NAFLD patients, and experimental mouse models consistently reveal significant alterations in gut microbial diversity and composition (Wan et al., 2019). An elevated Firmicutes/Bacteroidetes (F/B) ratio has been linked to NAFLD and obesity, as increased Firmicutes abundance appears to enhance energy harvest from the diet, leading to greater fat accumulation, oxidative stress, systemic inflammation, and aggravated liver injury (Magne et al., 2020). These gut microbiota structural changes can impair intestinal barrier function, promote endotoxin production, and disrupt the synthesis of hormones involved in nutrient and energy homeostasis (Monk et al., 2019). Therefore, a multi-target therapeutic strategy that addresses interconnected pathological factors, including insulin resistance, oxidative stress, inflammation, and gut-liver axis dysregulation, serves as an effective approach to alleviating and delaying the progression of NAFLD.

Paeoniflorin (PAF), a characteristic monoterpene glycoside derived from the roots of *Paeonia lactiflora*, has been demonstrated to possess multiple pharmacological activities, including antioxidant, anti-inflammatory, and neuroprotective effects (Tao et al., 2016; Ma et al., 2020; Gong P. et al., 2024). PAF demonstrates favorable pharmacokinetics and a high safety profile, characterized by rapid systemic absorption and minimal toxicity (Ou et al., 2025). PAF demonstrates broad therapeutic efficacy across multiple disease models, such as cardiac hypertrophy, Parkinson’s disease, and diabetic complications, primarily through potent antioxidant and anti-inflammatory mechanisms (Ren et al., 2023; Gong X. et al., 2024). PAF alleviates atherosclerosis primarily by modulating the gut microbiota-metabolite axis, which in turn improves blood lipids, reduces arterial inflammation, and restores intestinal barrier integrity (Xia et al., 2025). In a rat model of alcohol withdrawal, PAF alleviated anxiety-like behaviors concurrent with attenuated systemic inflammation and a beneficial shift in the gut microbiota, specifically enriching anti-inflammatory genera and depleting harmful bacteria (Wang et al., 2022a). Notably, PAF exhibits remarkable efficacy in ameliorating liver injury and regulating glucose and lipid metabolism (Ma et al., 2017; Zhao et al., 2017), providing a strong theoretical foundation for its application in preventing and treating obesity-related metabolic disorders. Existing studies confirm that PAF can significantly ameliorate hepatic lipid metabolism disorders and enhance energy expenditure in obese animal models by activating the AMPK signaling pathway and suppressing key metabolic enzymes such as fatty acid synthase (FAS) (Bi et al., 2023). However, it is not fully understood whether PAF’s protective role against NAFLD involves not only the regulation of the gut-liver axis but also a fundamental attenuation of oxidative stress, potentially mediated through the restoration of microbial homeostasis and intestinal barrier function.

These findings suggest that PAF may mitigate obesity-associated metabolic inflammation and oxidative stress via the gut microbiota-intestinal barrier-liver regulatory network. To test this hypothesis, the present study established a high-fat/high-sugar diet-induced NAFLD mouse model to systematically elucidate whether paeoniflorin (PAF) alleviates NAFLD through its antioxidant effects mediated by the gut microbiota–intestinal barrier–liver axis. By integrating histopathological analysis, oxidative stress markers, microbiome sequencing, metabolomics, and fecal microbiota transplantation, we aim to uncover the role of PAF in bridging gut microbial ecology and systemic oxidative balance, thereby providing solid experimental evidence for its potential as a therapeutic strategy against NAFLD.

## 2 Materials and methods

### 2.1 Materials and reagents

Paeoniflorin (purity ≥ 98%) was purchased from Aladdin Biochemical Technology (Shanghai, China). Antibodies against Superoxide Dismutase 1 (SOD1), and Superoxide Dismutase 2 (SOD2) were purchased from Huaan Biotechnology Co., Ltd. (Hangzhou, China). Secondary antibodies against rabbit were obtained from Sanying Biotechnology Co., Ltd. (Wuhan, China).

### 2.2 Animals

**FIGURE 1:**
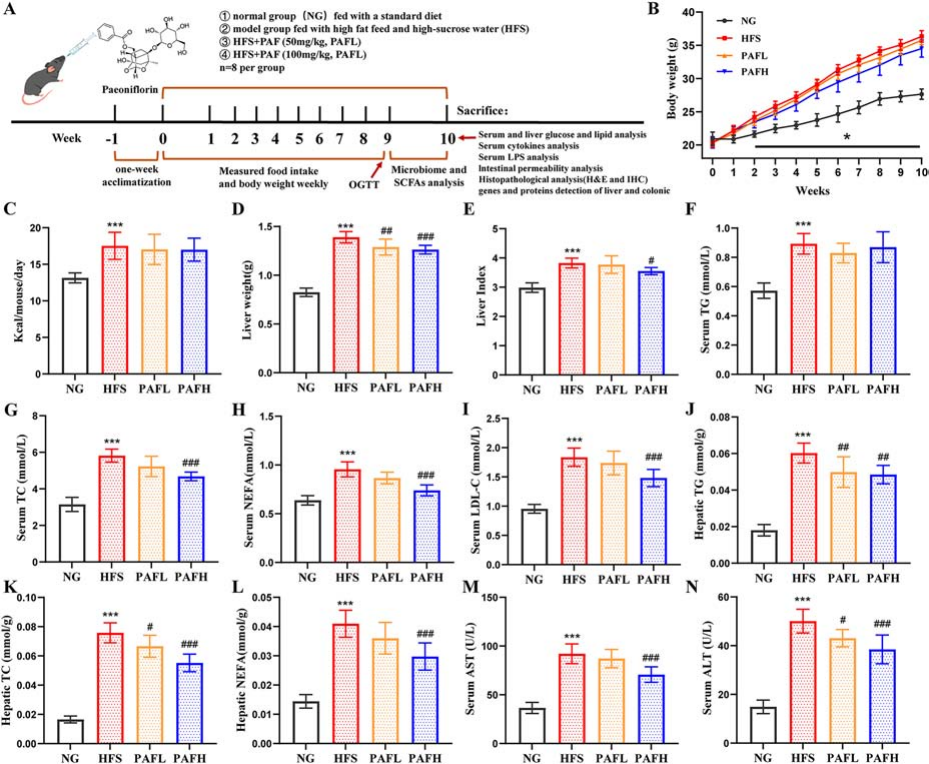
PAF supplementation improved lipid indices in NAFLD mice induced by HFS diet. **(A)** Schematic representation the experimental protocol used in this study, *n* = 8 pre group. **(B)** Body weight, **(C)** daily energy intake, **(D)** liver weight, **(E)** liver index. **(F,J)** triglyceride (TG), **(G,K)** total cholesterol (TC), **(H,L)** non-esterified fatty acid levels (NEFA) in serum and liver respectively. **(I)** low-density lipoprotein cholesterol (LDL-C) in serum. **(M,N)** serum aspartate aminotransferase (AST) and alanine aminotransferase (ALT) activities. NG, normal control group; HFS, high-fat/sucrose diet fed group; PAFL, paeoniflorin at low dosage (50 mg/kg/d); PAFH, paeoniflorin at high dosage (100 mg/kg/d). Data are expressed as means ± SD. Significance was determined by the ANOVA variance test, * HFS vs. NG, ^#^PAFL or PAFH vs. HFS. ^#^/*P < 0.05, ^##^*P* < 0.01, and^###^/***P < 0.001.

C57BL/6 mice, aged 8 weeks and weighing approximately 20 ± 2 g, were acquired from Heilongjiang Yuheng Veterinary Science and Technology Service Co., Ltd. All procedures were conducted with ethics approval (QMU-AECC-2022119) and mice were housed in an SPF facility at Qiqihar Medical University. After a 1-week acclimatization phase, the mice were randomly assigned to four groups (*n* = 8 per group). In the NG group, mice were fed a standard diet. In the model group (HFS) and the Paeoniflorin supplement group (PAF), mice were fed with a high-fat diet (D12492 formula, Shenyang Maohua Biotechnology Co., Ltd.) and high-sucrose water (drinking water supplemented with 30% sucrose). Additionally, the Paeoniflorin supplement group (PAFL and PAFH) received Paeoniflorin at 50 and 100 mg/kg/day via oral gavage, respectively, based on previous studies demonstrating efficacy in anti-oxidative and anti- inflammatory (Wang et al., 2022a). Meanwhile, the NG and HFS groups were administered an equivalent volume of normal saline. Mice were fed for 10 weeks with weekly monitoring of feeding conditions and weight recordings. An overnight fasting protocol was implemented 24 h before experiment termination. The following day, their fasted body weights were measured and fasting blood-glucose (FBG), and then euthanized by anesthesia. After the mice were fully anesthetized, liver, colon, and epididymal white adipose tissue (eWAT) were carefully dissected, weighed, snap-frozen in liquid nitrogen and stored at −80°C for RT-qPCR, western blot and ELISA experiments. Portions of the liver, colon and eWAT were fixed in a 4% paraformaldehyde solution for subsequent pathological analysis and experimentation. Fresh fecal samples were also collected and stored at −80°C for gut microbiota composition and short-chain fatty acids (SCFAs) analysis (Figure 1A).

### 2.3 Biochemical assays

Blood samples were collected from the orbital vein (4,000 rpm for 30 min at 4°C) for biochemical and metabolomics analysis. Serum biochemical indicators (TC, TG, NEFA, LDL-C, AST, and ALT) were determined by an automatic biochemical analyzer (Mindray BS-280, Shenzhen, China). The SOD and MDA were tested by commercial assay kits (Solarbio Bioscience & Technology Co., Ltd., China). The inflammatory factors, including tumor necrosis factor-α (TNF-α), interleukin-1β (IL-1β), interleukin-6 (IL-6), and lipopolysaccharide (LPS) levels and serum fasting insulin (FINS) were analyzed using ELISA kits (Beijing 4A Biotech Co., Ltd., China). The homeostasis model assessment parameter of insulin resistance (HOMA-IR) was calculated using the formula: HOMA-IR = [Plasma glucose (mmoL/L) × serum insulin (mIU/L)]/22.5. Samples were processed and analyzed in a randomized order using coded labels. The technicians conducting the assays were unaware of the corresponding experimental groups.

### 2.4 Oral glucose tolerance test

An oral glucose tolerance test (OGTT) was conducted during the 10th week of treatment, following established protocols. Mice were subjected to a 12-h fasting period before being administered an oral glucose solution at a dose of 2.0 g/kg of body weight. Blood samples were collected at 0, 30, 60, and 120 min post-glucose administration using a blood glucose meter (Accu-Chek, Roche, Switzerland) via tail blood collection. The area under the curve (AUC) represents glucose excursion during OGTT and is calculated using the trapezoidal rule (Xu et al., 2020).

### 2.5 Histology and immunohistochemistry

Liver and colon tissues were fixed in 4% paraformaldehyde, embedded in paraffin, sectioned, and stained with hematoxylin and eosin (H&E), and subsequently analyzed with light microscopy (Song et al., 2021). Immunohistochemistry assays were used to evaluate the expression of oxidative stress markers SOD1 and SOD2 in liver and intestinal tissues. Antigen retrieval was performed by heating the sections in citrate buffer (pH 6.0) at 121°C for 3 min. After cooling and PBS washing, endogenous peroxidase activity was blocked with 3% hydrogen peroxide for 15 min. Non-specific binding was blocked with 5% BSA for 30 min at room temperature. Sections were then incubated overnight at 4°C with primary antibodies against SOD1 (1:500) and SOD2 (1:800). After PBS washes, the sections were incubated with HRP-conjugated secondary antibodies for 1 h at room temperature. Immunoreactivity was visualized using DAB chromogen, followed by hematoxylin counterstaining. Finally, sections were dehydrated, cleared in xylene, and mounted for microscopic examination. All slides were coded with unique numerical identifiers by a researcher not involved in the assessment. The authors performing the analysis had no access to the group key until all evaluations were completed.

### 2.6 Quantitative real-time PCR

Total RNA from liver and colon samples was isolated using the Biozol reagent (Invitrogen, Carlsbad, CA, United States), as outlined in previous research (Sang et al., 2021). cDNA was synthesized by using a reverse transcriptase kit (TaKaRa, Beijing, China). Quantitative Real-Time PCR was conducted on qTOWER3G system (Analytik Jena, Germany), and Trans Start^®^ Green qPCR SuperMix (TransGen Biotech, Beijing, China) was used. The expression levels were determined according to the 2^–△△Ct^ method. Details about the primers can be found in Supplementary Table S1.

### 2.7 Short-chain fatty acids analysis

The fecal samples collected at the end of the 10-week intervention period and stored at −80°C until analysis. Fecal short-chain fatty acids (SCFAs) were extracted by homogenizing samples in methanol, followed by acidification and centrifugation. Analysis was performed on an Agilent 7890A gas chromatography (GC) system equipped with a DA-FFAP column and FID detector. Key parameters included: injector/detector temperatures of 250°C, nitrogen carrier gas at 30mL/min, split injection (50:1), and a temperature gradient from 80 to 180°C at 10°C/min. Analysis of SCFAs including acetic acid, propionic acid, butyric acid, isobutyric acid, and valeric acid, was performed at Biomarker Technologies Co., Ltd. (Beijing, China). Specific methodologies for detection are extensively outlined in earlier research (Wang et al., 2022b).

### 2.8 Untargeted metabolomics analysis

To elucidate the underlying mechanisms, we selected the PAFH group (hereafter the PAF group), NG group, and HFS group (*n* = 8 per group) for untargeted metabolomics and gut microbiota analysis, based on the superior NAFLD-alleviating efficacy of the PAFH group. The experimental process, which included serum extraction, original data collection, and bioinformatics analysis, was conducted by Beijing Baimaike Technology Co., Ltd.^1^ Metabolomics analysis was performed using the ultra-high performance liquid chromatography system, Waters Acquity I-Class PLUS, coupled with the high-resolution mass spectrometer, Waters Xevo G2-XS QTOF. Detailed procedures for data analysis were provided in Supplementary Methods.

### 2.9 Gut microbiota composition analysis

The full-length 16S rRNA genes were analyzed using third-generation sequencing on the PacBio sequencing platform provided by Biomarker Technologies Co., Ltd. (Beijing, China). Sequencing of marker genes was conducted using the SMRT Cell method, followed by filtering, clustering, and denoising of sequences through Circular Consensus Sequencing (CCS) (see text footnote 1).

### 2.10 Fecal microbiota transplantation

Fecal microbiota transplantation was performed following the methodology outlined earlier (Wang et al., 2022b). Male C57BL/6J mice, 6 weeks old (weighing 20.0 ± 2.0 g), were given a normal control (NG) diet and administered antibiotics (0.5 g/L vancomycin, 1.0 g/L ampicillin, 1.0 g/L metronidazole, and 1.0 g/L neomycin sulfate) to create conditions resembling a pseudo germ-free environment. The antibiotics were mixed daily in distilled water for the mice to drink. After a 2-week course of antibiotic therapy, the mice with depleted microbiota were randomly assigned to two distinct groups: MTHFS and MTPAF (*n* = 5 per group). These groups received microbiota transplants from mice that had been fed a HFS and PAF for 10 weeks, respectively. The preparation of donor samples for fecal microbiota transplantation was described previously (Wang et al., 2022b).

### 2.11 Statistical analysis

Statistical analyses were conducted using GraphPad Prism software (version 8.3.0, San Diego, California, United States). The quantitative data were expressed as mean ± SD. Differences among the three groups were assessed using one-way analysis of variance (ANOVA) followed by Tukey’s *post-hoc* test. A significance level of *P* < 0.05 was established.

## 3 Results

### 3.1 PAF treatment ameliorated HFS-induced metabolic disorders

Throughout the 10-week experiment (Figure 1A), the HFS group exhibited significantly greater body weight gain than the NG group (*P* < 0.05) (Figure 1B). Although the PAFL and PAFH group had a lower body weight gain rate than the HFS group, this difference had no significance (*P* > 0.05). There was no significant difference in energy intake among the HFS, PAFL, and PAFH groups (Figure 1C). In terms of hepatic parameters (Figures 1D,E), liver weight and liver index were significantly higher in the HFS group than in the NG group. Although PAFL treatment significantly reduced liver weight, it did not markedly affect the liver index, whereas PAFH treatment markedly reduced both parameters compared to the HFS group (*P* < 0.05). Serum and hepatic lipid levels (Figures 1F–L) differed significantly. The HFS group exhibited elevated serum TG, TC, NEFA, LDL-C, and hepatic TG, TC, NEFA relative to the NG group (*P* < 0.001). PAFL significantly reduced hepatic TG and TC, but did not markedly affect the other parameters. In contrast, PAFH treatment significantly reversed these abnormal increases, except for serum TG. Moreover, serum AST and ALT levels were significantly elevated in the HFS group compared to the NG group (*P* < 0.001). While PAFL treatment significantly lowered ALT levels without markedly affecting AST, PAFH administration notably reduced both enzyme activities compared to the HFS group (*P* < 0.001) (Figures 1M,N). These findings indicate PAF administration, particularly at the high dose (PAFH) providing comprehensive improvement by attenuating hepatic steatosis, dyslipidemia, and liver enzyme elevations induced by the HFS diet.

### 3.2 PAF treatment alleviates NAFLD by reversing lipid accumulation and regulating glycolipid metabolism genes expression

**FIGURE 2:**
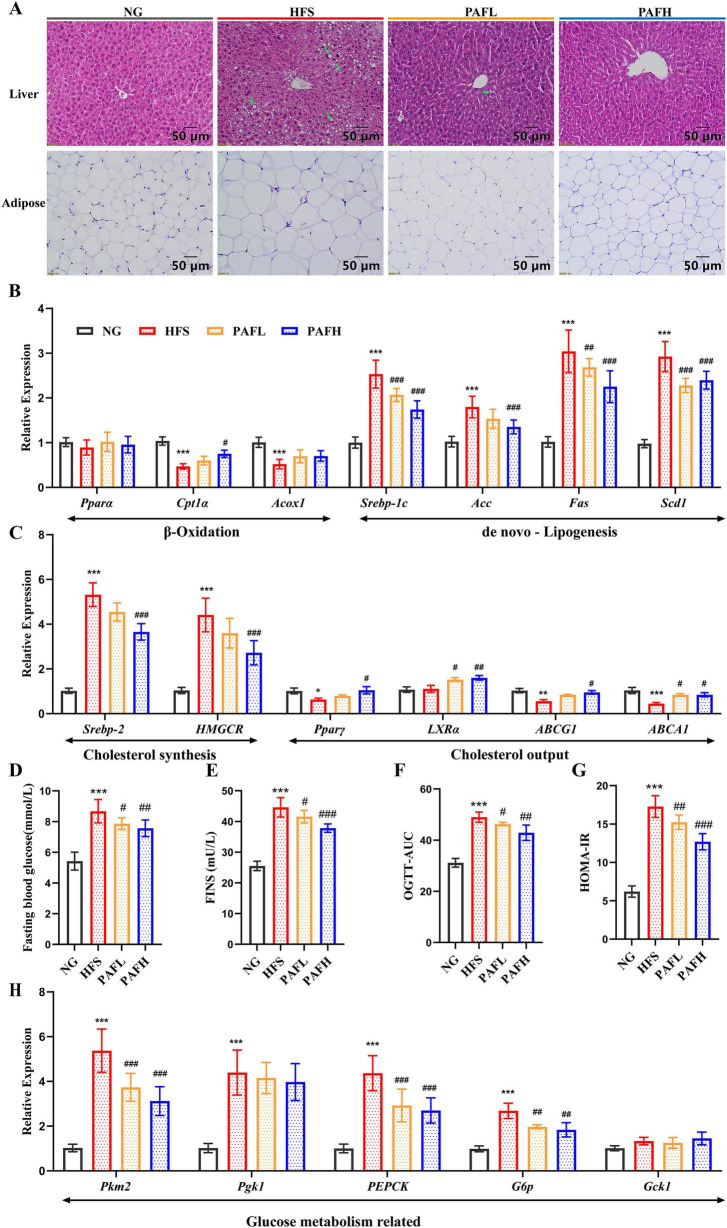
PAF attenuates lipid accumulation and modulates the expression of glycolipid metabolism-related genes. **(A)** H&E staining of mice livers and adipose. The length of the bar in the figure represents 50 μm. Green arrows showed the lipid droplets in liver **(B,C)** Gene expressions of lipid metabolism in the liver. **(D)** Fasting blood glucose, **(E)** serum fasting insulin (FINS) levels, **(F)** the area under the curve of oral glucose tolerance test (OGTT-AUC), **(G)** homeostasis model assessment parameter of insulin resistance, HOMA-IR, **(H)** gene expressions of glucose metabolism in the liver. NG, normal control group; HFS, high-fat/sucrose diet fed group; PAFL, paeoniflorin at low dosage (50 mg/kg/d); PAFH, paeoniflorin at high dosage (100 mg/kg/d). Data are expressed as means ± SD (*n* = 8 per group). Significance was determined by the ANOVA variance test, *HFS vs. NG, ^#^ PAFL or PAFH vs. HFS, ^#^/*P < 0.05, ^##**^P < 0.01, and ^###***^P < 0.001.

To characterize the histopathological changes in the liver and adipose tissue, H&E staining was performed. The results showed that HFS-fed mice exhibited typical features of non-alcoholic fatty liver disease including extensive microvesicular and macrovesicular steatosis, accompanied by mild hepatocyte swelling, disorganized lobular structure and mild inflammatory infiltration (Figure 2A). PAFL and PAFH intervention significantly attenuated these changes by reducing lipid accumulation, restoring cellular organization, and mitigating tissue injury, indicating amelioration of NAFLD-associated steatosis and hepatic damage. In the adipose tissue, the HFS group showed significantly increased cell diameter and loose intercellular spacing, indicating the marked adipocyte hypertrophy and consistent with the results of adipose tissue mass increased in HFS group compared with the NG (Supplementary Figure S1). PAF treatment at both high and low dosage demonstrated a substantial reduction in adipocyte size, with cellular morphology approaching that of the normal control, indicating effective attenuation of adipose tissue hypertrophy (Figure 2A).

To elucidate PAF’s regulation of glycolipid metabolism in NAFLD mice, we analyzed the hepatic expression levels of key genes associated with glycolipid metabolic pathways. When compared with the NG group, the HFS group exhibited significantly decreased expression of β-oxidation-related genes (*Cpt1*α, *Acox1*) and increased expression of *de novo* lipogenesis-related genes (*Srebp-1c*, *Acc*, *Fas*, *Scd1*) (*P* < 0.001) (Figure 2B). Conversely, both PAFL and PAFH treatments partially reversed these alterations toward NG levels. PAFH exerted more pronounced effects, particularly on genes of *Cpt1*α and *Acc*. Notably, no significant inter-group difference was observed in *Ppar*α expression across the four groups. Collectively, these results highlight that PAF regulates hepatic lipid catabolism by promoting catabolism while inhibiting lipid synthesis in a dose-dependent manner. Regarding cholesterol metabolism (Figure 2C), compared with the NG group, the expression of cholesterol synthesis genes (*Srebp-2* and *HMGCR*) was higher in the HFS group, while the expression of cholesterol output genes (*Ppar*γ, *ABCG1*, and *ABCA1*), which play crucial roles in cholesterol reverse transport (RCT) was significantly lower (*P* < 0.05). PAFL treatment slightly reduced synthesis gene expression (*Srebp-2* and *HMGCR*) with no significant compared with HFS group, but significantly enhanced cholesterol output genes expression such as *LXR*α and *ABCA1*. In contrast, PAFH treatment more effectively restored these changes, significantly suppressing *Srebp-2* and *HMGCR* expression while enhancing the expression of cholesterol output genes (*Ppar*γ, *LXR*α, *ABCG1*, and *ABCA1*), demonstrating a stronger regulatory role in cholesterol metabolism.

Regarding glucose metabolism, the fasting blood glucose, FINS, OGTT-AUC and HOMA-IR levels were significantly higher in the HFS group compared to the NG group (*P* < 0.001) (Figures 2D–G), indicating significant insulin resistance and impaired glucose homeostasis in NAFLD mouse induced by the HFS diet. In contrast, both PAFL and PAFH group had lower levels of these parameters compared to the HFS group (*P* < 0.01), with PAFH demonstrating a more pronounced improvement, suggesting a dose-dependent amelioration of insulin resistance and glucose intolerance. Treatment with PAFL or PAFH normalized the aberrant expression of glucose metabolism-related genes induced by HFS, including *Pkm2*, *PEPCK*, and *G6p*, while the expression of *Pgk1* and *Gck1* remained unchanged compared with HFS group (Figure 2H). Collectively, these results suggest that both PAFL and PAFH supplementation modulate the gene expression related to lipid and glucose metabolism and improve glucose-related physiological parameters in NAFLD mouse induced by HFS diet, with more pronounced effects observed in the PAFH group.

### 3.3 PAF treatment enhanced antioxidant capacity and suppressed pro-inflammatory cytokines

**FIGURE 3:**
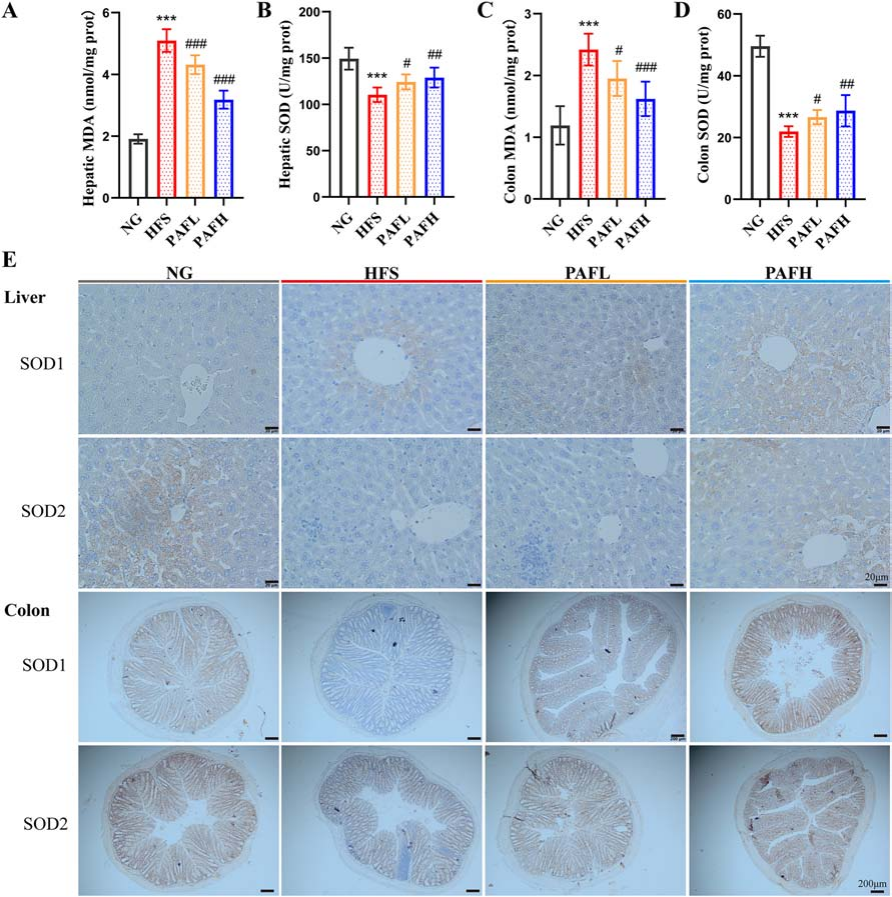
PAF exerts antioxidant effects in NAFLD mice by reducing oxidative stress and enhancing antioxidant enzymes activity. **(A–D)** The oxidative stress and antioxidant indices in mice subjected to HFS. Significance was determined by the ANOVA variance test * HFS vs. NG, ^#^ PAFL or PAFH vs. HFS, ^#^*P* < 0.05, ^##^*P* < 0.01, and ^###^/***P < 0.001. **(E)** IHC for the distribution of adhesion molecules SOD1 and SOD2 in liver and colon tissues of mice. The bar in the liver figure represents 20 μm and the bar in the colon figure represents 200 μm. NG, normal control group; HFS, high-fat/sucrose diet fed group; PAFL, paeoniflorin at low dosage (50 mg/kg/d); PAFH, paeoniflorin at high dosage (100 mg/kg/d). Data are expressed as means ± SD (*n* = 8 per group).

To evaluate the antioxidant capacity and anti-inflammatory of PAF in NAFLD mice, serum and target tissues levels of lipid peroxidation biomarkers (MDA), antioxidant enzyme activities (SOD1, SOD2) and pro-inflammatory cytokines were measured. The antioxidant activity of PAF was assessed in serum (Supplementary Figures S2A,B), liver, and colon tissues (Figures 3A–D). The HFS group exhibited elevated MDA levels and reduced SOD activity in both kind of tissues and serum, indicating oxidative stress states in NAFLD mice. PAFL treatment decreased MDA levels and enhanced SOD activity in these tissues (*P* < 0.05), demonstrating improved antioxidant capacity. PAFH treatment produced a more pronounced effect, further reducing MDA and elevating SOD activity to a greater extent, indicating a dose-dependent enhancement of antioxidant defense. Subsequently, immunohistochemistry (IHC) was employed to further analyze the expression of SOD1 and SOD2 in liver and colon tissues (Figure 3E). In the HFS group, the expression levels of SOD1 and SOD2 were markedly decreased in both liver and colon tissues. PAFL and PAFH treatment significantly upregulated the expression of SOD1 and SOD2 in these tissues. These results indicated that PAF demonstrated potent antioxidant activity in NAFLD mice by mitigating lipid peroxidation and boosting endogenous antioxidant defenses across systemic and tissue levels.

**FIGURE 4:**
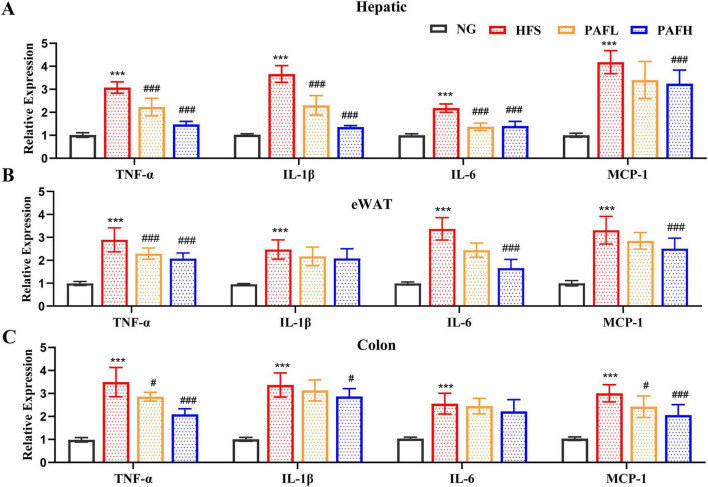
PAF effectively ameliorates inflammatory by downregulating pro-inflammatory genes expression. **(A–C)** The gene expression of inflammatory factors at the mRNA level in liver **(A)**, epididymal white adipose tissue, eWAT **(B)**, and colon **(C)**. NG, normal control group; HFS, high-fat/sucrose diet fed group; PAFL, paeoniflorin at low dosage (50 mg/kg/d); PAFH, paeoniflorin at high dosage (100 mg/kg/d). Data are expressed as means ± SD (*n* = 8). Significance was determined by the ANOVA variance test * HFS vs. NG, ^#^PAFL or PAFH vs. HFS, ^#^*P* < 0.05, and ^###^/***P < 0.001.

Fat accumulation and oxidative stress jointly contribute to increased inflammation in NAFLD, here we detected the pro-inflammatory cytokines in serum and the relative gene expression in different tissues. Compared with the NG group, the HFS group exhibited chronic inflammation characterized by increased levels of pro-inflammatory cytokines (TNF-α, IL-1β, IL-6, and MCP-1) in serum. Notably, supplementation with PAFL or PAFH significantly inhibited the development of chronic inflammation compared to the HFS group, except for IL-1β (Supplementary Figures S2C–F). The anti-inflammatory effect of PAF was further corroborated by the decreased relative mRNA expression levels of pro-inflammatory cytokines (TNF-α, IL-1β, IL-6, MCP-1) in liver, adipose, and colon tissues respectively (Figures 4A–C). Compared to the HFS group, the PAFL group showed a significant reduction in the expression of most pro-inflammatory cytokines (*P* < 0.05), except for MCP-1 in liver and adipose, and IL-1β and IL-6 in adipose. In contrast, PAFH treatment resulted a more comprehensive suppression, decreasing the expression levels of TNF-α, MCP-1 across all three tissues, as well as reducing IL-1β in liver and colon tissues, and IL-6 in liver and eWAT tissues, indicating a stronger anti-inflammatory activity of PAFH.

### 3.4 PAF treatment reshaped the serum metabolome and modulated key metabolic pathway in NAFLD mice

**FIGURE 5:**
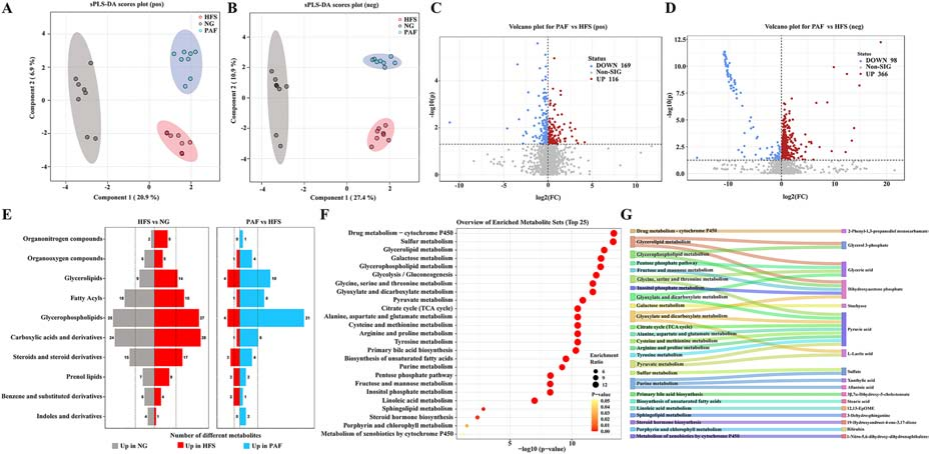
The serum metabolic profile altered by PAF in HFS- fed mice. **(A,B)** Score plot of the sPLS- DA model in positive and negative ion modes, **(C,D)** differential metabolites in serum identification in HFS and PAF groups in positive and negative ion modes, **(E)** the number of differential metabolites was annotated and classified based on the HMDB changed by HFS and the treatment of PAF, **(F)** the enrichment metabolic pathway of fecal altered metabolites by Kyoto encyclopedia of genes and genomes (KEGG) analysis. **(G)** The Sankey plot illustrates the relationship between the regulated metabolites and the enriched pathway. NG, normal control group; HFS, high-fat/sucrose diet fed group; PAF, paeoniflorin at high dosage (100 mg/kg/d). FC, fold change, *n* = 8 per group.

Based on the superior efficacy in alleviating NAFLD observed in the PAFH group, it was selected for further studies to elucidate its mechanisms and hereafter referred to as the PAF group. Serum metabolites may serve as key mediators in PAF’s anti-NAFLD pathway, offering crucial insights to elucidate the underlying mechanisms. To uncover these novel biomarkers, untargeted metabolomics profiling was applied to serum samples. The results of sPLS-DA demonstrated distinct differentiations in both positive and negative ion modes among the three groups (Figures 5A,B). To further enhance the analysis of changes in serum metabolites, a volcano plot was utilized to illustrate the overall differences. Metabolites with a Variable Importance in Projection (VIP) > 1, *P* < 0.05, and a fold change (FC) of at least 1 were regarded as being significantly modified by PAF supplementation. Compared to the NG group, the HFS group showed an upregulation of 528 metabolites and a downregulation of 311 metabolites in the positive ionization mode, while in the negative ionization mode, there was an upregulation of 502 metabolites and a downregulation of 600 metabolites (Supplementary Figures S3A,B). Additionally, in the PAF vs. HFS comparison, a total of 116 metabolites were upregulated and 169 metabolites were downregulated in the positive ionization mode, and 366 metabolites were upregulated and 98 were downregulated in the negative ionization mode in PAF compared to the HFS group (Figures 5C,D). To characterize the metabolic regulatory effects of PAF, we analyzed metabolite abundance changes via metabolomic heatmaps (Supplementary Figures S2C,D). Samples from the same group clustered tightly, with the PAF group’s metabolic signature clearly separated from HFS in both positive and negative ion mode. PAF treatment elicited extensive and significant changes in the serum metabolome, highlighting its role in systemic metabolic modulation during NAFLD alleviation.

The Human Metabolome Database (HMDB) was utilized to annotate and classify the differential metabolites (Figure 5E). When comparing HFS to NG, various metabolite classes such as Glycerophospholipids, Carboxylic acids and derivatives, Fatty Acyls, Steroids and steroid derivatives, and Glycerolipids, showed changes in the number of differentially abundant metabolites. The PAF exerted an influence on a wide range of metabolites, predominantly targeting Glycerophospholipids, Glycerolipids, and Fatty Acyls. Furthermore, a comparison between the HFS group and the PAF group demonstrated that KEGG pathway enrichment analysis identified the top 25 specific pathways with significant alterations (Figure 5F). The bubble map analysis illustrated the regulatory changes following PAF treatment in pathways involved in core energy and nutrient metabolic pathways, such as Drug metabolism-cytochrome P450, Sulfur metabolism, Glycerolipid metabolism, Galactose metabolism, Glycerophospholipid metabolism, Glycolysis/Gluconeogenesis, Glycine, serine and threonine metabolism, Glyoxylate and dicarboxylate metabolism, Pyruvate metabolism, and the Citrate cycle (TCA cycle). These pathways are closely associated with lipid accumulation, energy dysregulation, and oxidative stress in NAFLD. Moreover, PAF modulated some central energy metabolism and secondary metabolite pathways such as Glycine, serine and threonine metabolism, Purine metabolism and Primary bile acid biosynthesis, facilitating the recovery of metabolic equilibrium. To link the enriched metabolic pathways with specific regulated metabolites by PAF, the pathway-metabolite association Sankey plot was constructed (Figure 5G). The metabolites like Glycerol 3-phosphate, Dihydroxyacetone phosphate, and Pyruvic acid which are critical intermediates in glycolysis and energy production was significantly regulated by PAF to restore the energy homeostasis. Metabolites such as Glyceric acid and Glycerol 3-phosphate which also served as membrane lipid precursors were modulated, reflecting PAF’s role in alleviating lipid accumulation. Notably, individual metabolites such as Pyruvic acid, Dihydroxyacetone phosphate and Glycerol 3-phosphate were involved in multiple pathways, highlighting the pleiotropic regulatory effects of PAF on interconnected metabolic networks. These findings suggest that PAF extensively reshapes the serum metabolome, primarily by modulating core pathways related to lipid and energy metabolism and regulating key intermediates, thereby promoting systemic metabolic homeostasis in NAFLD.

### 3.5 PAF administration alleviates HFS-induced intestinal barrier damage

**FIGURE 6:**
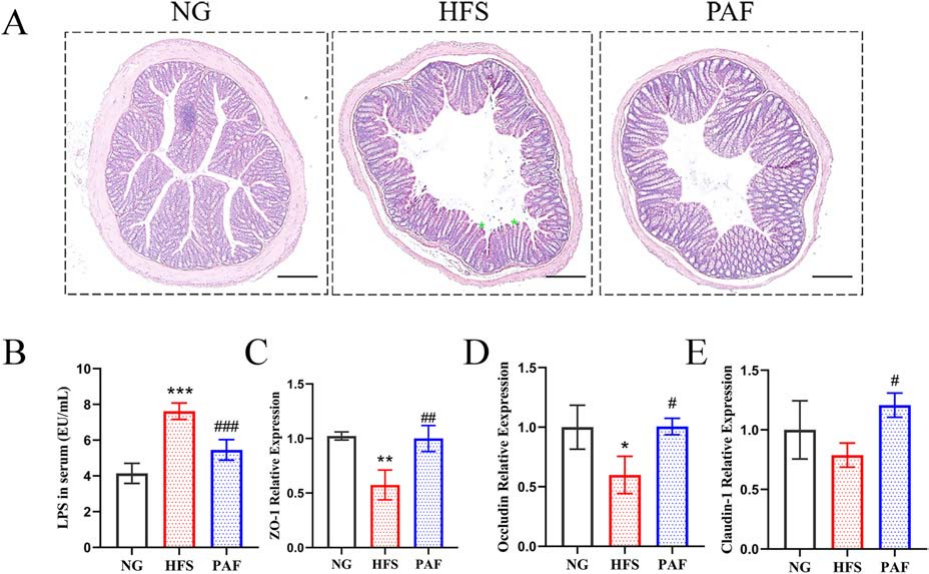
PAF administration alleviates HFS-induced intestinal barrier damage. **(A)** H&E staining of colon tissue sections, the scale bar represents 200 μm. Asterisks showed the structural alterations in colon. **(B)** Assessment of serum LPS level across experimental group, **(C–E)** qRT-PCR determination of tight junction genes ZO-1 **(C)**, Occludin **(D)** and Claudin-1 **(E)** expression in colon tissues. NG, normal control group; HFS, high-fat/sucrose diet fed group; PAF, paeoniflorin at high dosage (100 mg/kg/d). Values are expressed as mean ± SD in each group, * HFS vs. NG, ^#^ PAF vs. HFS, ^#^/*P < 0.05,^##^/**P < 0.01, and ^###^/***P < 0.001.

Increased intestinal permeability serves as a key mechanism that allows gut-derived bacterial products to reach the liver, thereby promoting inflammation, oxidative stress, and metabolic dysregulation in NAFLD. This study analyzed histopathological sections and the expression of genes related to intestinal permeability to assess the impact of PAF on intestinal barrier function during the alleviation of NAFLD. Compared with the NG group, the HFS group showed marked intestinal villous abnormalities, including disorganization, shortening, and compression, consistent with metabolically induced mucosal injury. In contrast, the PAF treatment preserved near-normal tissue architecture, suggesting that PAF effectively alleviates HFS-induced intestinal structural impairment (Figure 6A). Serum LPS levels reflect intestinal barrier integrity, as the intact epithelial tight junctions of intestinal cells prevent LPS translocation into circulation. The HFS group exhibited significantly elevated serum LPS levels than the NG group (*P* < 0.001), indicating impairment of the intestinal barrier dysfunction (Figure 6B). PAF treatment significantly reduced serum LPS levels compared to the HFS group, implying an improvement in intestinal barrier integrity. Accordingly, the mRNA expression of tight junction proteins were analyzed in colonic tissues (Figures 6C–E). In contrast to the HFS group, which showed reduced expression of ZO-1 and Occludin, the PAF group restored the expression of ZO-1, Occludin, and Claudin-1 relative to NG levels. These results suggest that PAF counteracts HFS-induced intestinal barrier damage by rescuing the expression of key tight junction proteins, leading to enhanced barrier integrity.

### 3.6 PAF treatment restructured the gut microbiota in NAFLD

**FIGURE 7:**
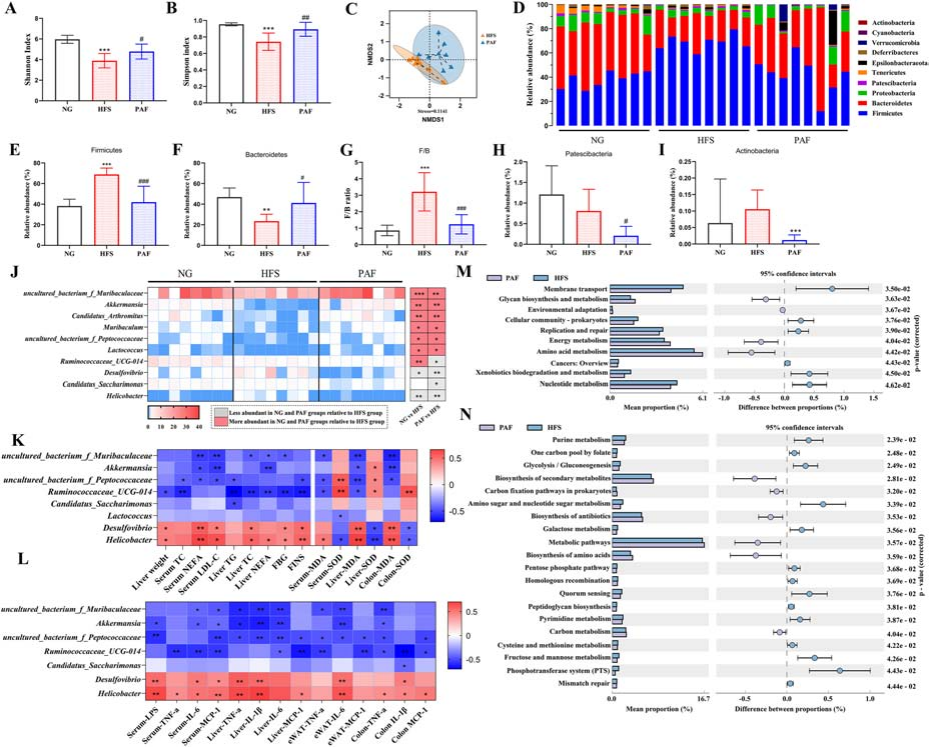
PAF modified bacterial composition in HFS-induced NAFLD mice. **(A,B)** α-Diversity of Shannon **(A)** and Simpson index **(B)** among groups. **(C)** Non-metric multidimensional scaling (NMDS) result based on Bray Curtis algorithm. **(D)** Changes of the composition of the gut microbiota at phylum taxa level, **(E–I)** significantly changes (*P* < 0.05) of the composition of the gut microbiota at phylum taxa level. **(J)** Significantly changes of the composition of the gut microbiota at genera taxa. **(K)** Heatmap of Spearman’s correlation between gut microbiota at genus level and biochemical indexes. **(L)** Correlation of gut microbiota with inflammatory factors and oxidative stress indicators at the genus level. **(M,N)** Differential KEGG pathways between the PAF and HFS groups identified using PICRUSt2 prediction at level 2 **(M)** and level 3 **(N)**. NG, normal control group; HFS, high-fat/sucrose diet fed group; PAF, paeoniflorin at high dosage (100 mg/kg/d). *P < 0.05, **P < 0.01. * HFS vs. NG, ^#^PAF vs. HFS, ^#^/*P < 0.05,^##^/**P < 0.01, and ^###^/***P < 0.001, *n* = 8 per group.

Gut microbiota dysbiosis increases intestinal permeability, triggering the translocation of bacterial products to the liver. This process promotes hepatic inflammation, oxidative stress, and metabolic dysfunction, ultimately driving the initiation and progression of NAFLD. To investigate the shifts in microbial composition and evaluate the potential dominance of specific microbiome communities in response to PAF, we analyzed compositional shifts via 16S rRNA sequencing. Firstly, α-diversity analysis (Shannon/Simpson indices) revealed significantly reduced microbial richness and evenness in HFS-fed mice compared with NG controls (*P* < 0.001). PAF treatment restored α-diversity to levels comparable to NG group (Figures 7A,B), indicating its capacity to restructure gut microbial communities. For the β-diversity analysis, non-metric multidimensional scaling (NMDS) based on Bray-Curtis distances was used. The results revealed a distinct separation in the gut microbiota composition among the NG, HFS, and PAF groups (Supplementary Figure S4A). Moreover, a significant difference was detected between the HFS and PAF groups (*P* < 0.004, *R* = 0.424, Stress = 0.1141) (Figure 7C).

We profiled gut microbiota composition by comparing dominant phyla and genera across groups, revealing significant HFS- and PAF-induced restructuring. At the phylum level (Figure 7D), Firmicutes, Bacteroidetes, Proteobacteria, Patescibacteria, and Tenericutes were the five most abundant phyla, comprising 95% of total gut bacteria across the groups. In comparison to the NG group, the HFS group increased the relative abundance of Firmicutes and the Firmicute/Bacteroidetes (F/B) ratio, while decreased the abundance of Bacteroidetes, Proteobacteria and Tenericutes (*P* < 0.05) (Figures 7E–G and Supplementary Figures S4B,C). Conversely, PAF supplementation reversed the abundance of Bacteroidetes, Firmicutes, and the F/B ratio. Furthermore, compared to the HFS group, PAF supplementation led to a reduction in the abundance of Patescibacteria and Actinobacteria (*P* < 0.05) (Figures 7H,I).

Among the top 15 genera, 10 were significantly altered by the HFS diet and PAF treatment (Figure 7J). Compared to NG group, HFS feeding reduced the abundances of certain genera, including *uncultured_bacterium_f_Muribaculaceae*, *Akkermansia*, *Candidatus_Arthromitus*, *Muribaculum*, *uncultured_bacterium_f_Peptococcaceae*, *Lactococcus*, and *Ruminococcaceae_UCG-014*, whereas increased *Desulfovibrio* and *Helicobacter* significantly (*P* < 0.05). Notably, PAF supplementation restored the relative abundances of these microorganisms at the genus level. Specifically, the relative abundance of *Akkermansia* in the HFS group was only 0.019%, but increased to 2.54% following PAF treatment.

Correlations between significantly altered genera and indices related to glycolipid metabolism, oxidative stress, and inflammatory cytokines were visualized in heatmaps (Figures 7K,L). The abundance of *uncultured_bacterium_f_Muribaculaceae* and *Akkermansia* (enriched by PAF intervention) exhibited significant negative correlations with serum NEFA, LDL-C levels, liver NEFA, and MDA levels in both liver and colon tissues (*P* < 0.05). Additionally, *Akkermansia* showed significant positive correlations with SOD levels in the liver (*P* < 0.05). The relative abundance of *Ruminococcaceae_UCG-014* was significantly negatively correlated with most glucolipid metabolic parameters and MDA levels in serum, liver, and colon tissues, but showed positive correlations with SOD levels in both serum and liver (Figure 7K). *Desulfovibrio* and *Helicobacter* showed positive correlations with most glucolipid metabolic parameters and MDA levels in serum, liver, and colon tissues. Additionally, negative correlations were observed between *uncultured_bacterium_f_Muribaculaceae, Akkermansia, uncultured_bacterium_f_Peptococcaceae* and levels of inflammatory cytokines in serum, and the gene expression of inflammatory cytokines in liver, adipose, and colon tissues (*P* < 0.05) (Figure 7L). In contrast, *Desulfovibrio* and *Helicobacter* showed positive correlations with most inflammatory cytokines in serum, and the gene expression of inflammatory cytokines in different tissues. The abundances of *Candidatus_Arthromitus* and *Muribaculum* showed no significant correlations with any of the measured indicators (*P* > 0.05) (Supplementary Figure S5).

To evaluate PAF-induced functional changes in gut microbiota, we employed Picrust2 to predict the functional profiles and mapped to KEGG pathways. At the second level of the KEGG analysis, the HFS diet significantly altered 27 functional pathways in comparison with the NG group (Supplementary Figure S6). In contrast, PAF treatment reversed 10 of these dysregulated functional categories, notably Membrane transport, Energy metabolism, Amino acid metabolism, and Nucleotide metabolism, all associated with substance and energy metabolism (Figure 7M). A more detailed analysis at the third level indicated that PAF significantly downregulated Glycolysis/Gluconeogenesis, Galactose metabolism, and Fructose and mannose metabolism. Meanwhile, it enhanced Biosynthesis of secondary metabolites, Biosynthesis of antibiotics, Biosynthesis of amino acids, Metabolic pathways, and Carbon metabolism (Figure 7N). Collectively, these findings highlight the role of PAF in reshaping microbial functional pathways linked to metabolism and environmental adaption in response to HFS induced NAFLD in mice.

### 3.7 PAF treatment elevated microbiota-derived fecal SCFAs

**FIGURE 8:**
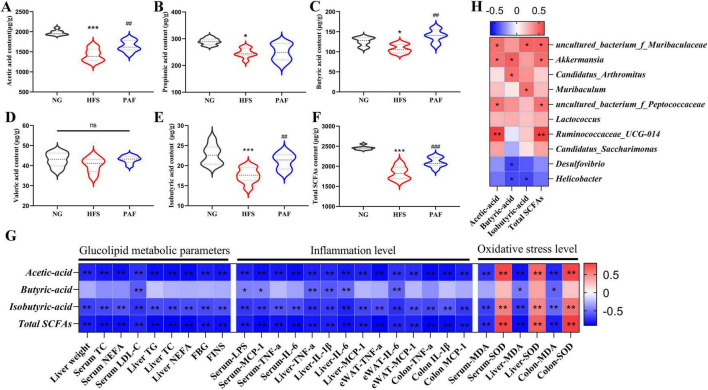
Effects of PAF supplementation on SCFAs production. **(A)** Acetic acid, **(B)** Propionic acid, **(C)** Butyric acid, **(D)** Valeric acid, **(E)** Isobutyric acid and **(F)** total SCFAs of fecal contents. **(G)** Correlation between the SCFAs and glucolipid metabolic parameters, inflammation level and oxidative stress level. **(H)** Correlation between the SCFAs and gut microbiota. NG, normal control group; HFS, high-fat/sucrose diet fed group; PAF, paeoniflorin at high dosage (100 mg/kg/d). Data are expressed as means ± SD (*n* = 8), Significance was determined by the ANOVA variance test, * HFS vs. NG, #PAF vs. HFS, ns, no significance, *P < 0.05, ^##^/**P < 0.01, and ^###^/***P < 0.001.

Reduced gut microbiota-derived short-chain fatty acids (SCFAs) contribute to NAFLD progression by impairing intestinal barrier function and hepatic metabolism, thereby promoting inflammation and oxidative stress. We analyzed the SCFAs content in feces and revealed PAF’s modulation of intestinal metabolism (Figure 8). HFS feeding significantly reduced fecal acetate, propionate, butyrate, isobutyrate and total SCFAs compared with NG group (*P* < 0.05) (Figures 8A–F). However, PAF treatment significantly increased the levels of acetic acid, butyric acid, isobutyric acid, and total SCFAs in the feces compared to the HFS group (*P* < 0.05). There was no significant difference in the content of valeric acid among the three groups (*P* > 0.05). These results showed that PAF alleviates HFS-induced depletion of beneficial SCFAs in the gut, highlighting a microbiota-mediated metabolic mechanism underlying its anti-NAFLD effect.

To further explore the correlations between SCFAs and glucolipid metabolism, oxidative stress, inflammation, and dominant genera, we constructed Spearman’s correlation heatmap (Figures 8G,H). As illustrated, acetic acid, isobutyric acid, and total SCFAs demonstrated significant correlations with glucolipid metabolic parameters, oxidative stress, and inflammation levels. The content of butyric acid exhibited significant negative correlations with serum LDL-C, LPS, and MCP-1 levels, as well as with TNF-α and IL-1β gene expression in the liver, and with IL-6 levels in both liver and eWAT, in addition to MDA levels in liver and colon tissues. Furthermore, acetic acid and total SCFAs were significantly positively correlated with the relative abundance of *uncultured_bacterium_f_Muribaculaceae*, *Akkermansia*, and *Ruminococcaceae_UCG-014*. Butyric acid showed significant positive correlations with the relative abundance of *Akkermansia* and *Candidatus_Arthromitus*, while exhibiting negative correlations with *Desulfovibrio* and *Helicobacter*. The results suggest that SCFAs restored by PAF functionally link gut microbiota remodeling to improved host pathophysiology in NAFLD, such as reduced lipid deposition, inflammation, and oxidative stress.

### 3.8 Fecal microbiota transplantation from PAF-treated mice ameliorates metabolic disorders in HFS-fed mice

**FIGURE 9:**
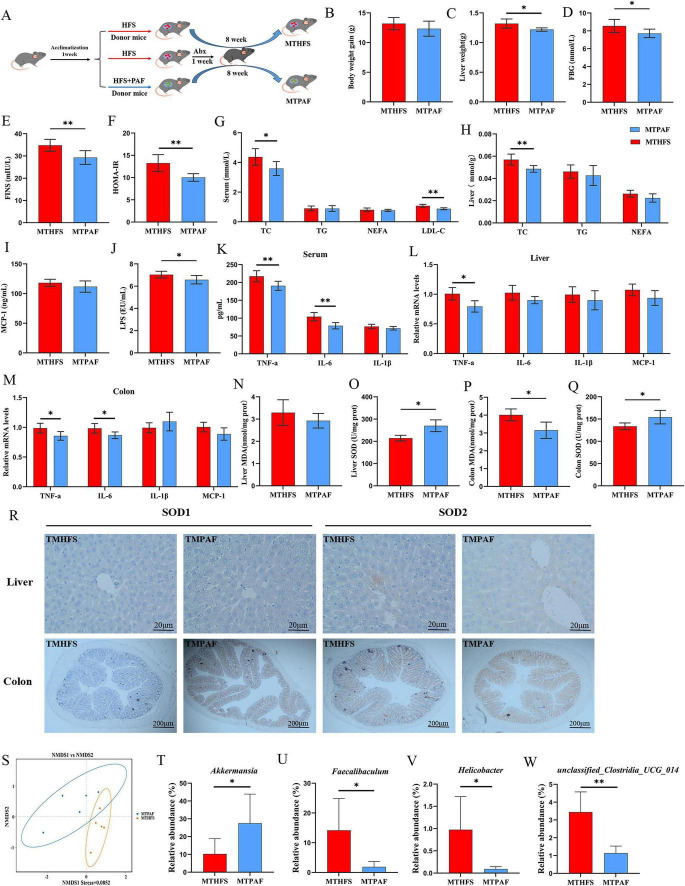
Transplantation of feces from mice treated with PAF improved the NAFD symptoms of HFS-fed mice. **(A)** The flow chart of the fecal microbiota transplantation (FMT) experiment. **(B)** Body weight gain. **(C)** Liver weight. **(D)** Fasting blood glucose (FBG). **(E)** Fasting insulin (FINS). **(F)** Homeostasis model assessment of insulin resistance (HOMA-IR). **(G)** Serum levels of lipids. **(H)** Levels of lipids in liver. **(I)** The level of MCP-1 in serum. **(J)** The level of LPS in serum. **(K)** The serum levels of inflammatory factors. **(L,M)** Relative mRNA expression levels of inflammatory factors genes. **(N–Q)** The levels of oxidative stress in the liver and colon. **(R)** IHC for the distribution of adhesion molecules SOD1 and SOD2 in liver and colon tissues of mice. **(S)**β-Diversity analysis of gut microbiota used non-metric multidimensional scaling (NMDS). **(T–W)** Significantly changes of the gut microbiota at genera taxa. MTHFS, the recipient mice that were fed a high-fat/sucrose diet and received fecal microbiota from HFS-fed donors. MTPAF, he recipient mice that were fed a high-fat/sucrose diet and received fecal microbiota from PAF treated donors. Data are the mean ± SD, *P < 0.05, and **P < 0.01.

To determine if PAF’s metabolic benefits require gut microbiota modulation, we performed fecal microbiota transplantation (FMT) from donor mice fed HFS with or without PAF into HFS-fed recipients (Figure 9A). Following 8-week FMT, liver weight was notably decreased in recipients of PAF-microbiota (MTPAF) mice compared to HFS-microbiota (MTHFS) mice (Figure 9C), although there were no significant differences in final body weight (Figure 9B). Moreover, MTPAF recipient mice exhibited lower levels of FBG, FINS, and HOMA-IR compared to MTHFS mice (Figures 9D–F). Serum lipid metabolism indexes revealed significant decreases in TC and LDL-C levels (*P* < 0.05), with parallel hepatic TC reduction, while serum and hepatic TG and NEFA remained unchanged between MTPAF mice and MTHFS mice (*P* > 0.05) (Figures 9G,H).

FMT from PAF-treated donors significantly reduced serum LPS, TNF-α, and IL-6 in MTPAF mice compared with MTHFS (*P* < 0.05) (Figures 9I–K). The level of MCP-1 and IL-1β also exhibited decreasing trends in MTPAF mice, though the difference was not statistically significant. Additionally, MTPAF mice exhibited significantly reduced TNF-α expression at mRNA level in both hepatic and colon tissues comparison to MTHFS mice, with concurrent downregulation of colonic IL-6 (Figures 9L,M). Collectively, these findings confirm that microbiota remodeling mediates systemic inflammation alleviation in NAFLD. Furthermore, to evaluate MTPAF’s impact on antioxidant capacity, we assessed serum, hepatic, and colonic oxidative stress biomarkers. The levels of MDA and SOD did not show significant differences between the MTHFS and MTPAF groups in serum (Supplementary Figure S7). However, MTPAF mice exhibited elevated SOD activity in hepatic and colonic tissues and reduced MDA levels in colonic tissues compared with MTHFS mice (*P* < 0.05) (Figures 9N–Q). The IHC results revealed significantly elevated SOD1 and SOD2 expression in colonic tissues, with no significant change in the liver tissues in the MTPAF group compared to MTHFS (Figure 9R).

To ascertain whether gut microbiota is causally linked to the beneficial effects of PAF on NAFLD, 16S rRNA sequencing was carried out in the FMT. There was no significant difference in Shannon index and the Simpson index between the MTHFS and MTPAF groups (Supplementary Figure S8). The NMDS analysis revealed a separation trend between the gut microbiota compositions of the two groups, suggesting that there were differences in the overall microbiota structure between MTHFS and MTPAF mice (Figure 9S). At the phylum level, when compared with the MTHFS group, the relative abundances of Firmicutes and Bacteroidetes in MTPAF mice were lower; however, these differences did not reach statistical significance (*P* > 0.05) (Supplementary Figures S9A,B). In contrast, the MTPAF group significantly decreased the abundance of Actinobacteriota and increased abundance of Verrucomicrobiota (Supplementary Figures S9C,D). At the genus level, MTPAF intervention induced differential abundance of key gut microbiota taxa. Specifically, MTPAF recipients exhibited significantly higher abundances of *Akkermansia*, but lower abundances of *Faecalibaculum, unclassified_Clostridia_UCG_014* and *Helicobacter* compared to MTHFS (Figures 9T–W). These results suggest that PAF-induced modulation of gut microbiota alleviates NAFLD and related disorders in HFS-fed mice.

## 4 Discussion

Obesity is the most significant risk factor for NAFLD in humans. The high-fat/high-sucrose (HFS) diet induced obese mouse model is widely employed in NAFLD research (Kim et al., 2022; Jouenne et al., 2025) because it effectively recapitulates key features of human metabolic syndrome and NAFLD progression, including insulin resistance, dyslipidemia, hepatic steatosis, low-grade inflammation, and gut microbiota dysbiosis. The present study elucidates a novel gut-liver axis mechanism underpinning paeoniflorin (PAF)-mediated protection against HFS diet-induced NAFLD. While prior studies indicate paeoniflorin alleviates non-alcoholic steatohepatitis (Ma et al., 2017; Ma et al., 2020), our study systematically combines histopathology, oxidative stress markers, serum metabolomics, 16S rRNA sequencing, and fecal microbiota transplantation (FMT). Although antioxidant effects of PAF have been reported (Guo et al., 2025), our research specifically delineates how PAF alleviates NAFLD-associated oxidative stress by enhancing systemic and tissue-specific antioxidant capacity (SOD activity, MDA reduction) and illustrates how this effect is coupled with microbial remodeling. The correlation and FMT data together establish a clear cause-effect pathway from PAF intake to microbial restructuring, then to metabolite change (SCFAs), and finally to host antioxidant/anti-inflammatory response. Crucially, fecal microbiota transplantation (FMT) experiments confirmed the causal role of microbial restructuring, as recipients of PAF-modulated microbiota recapitulated key metabolic improvements, including reduced hepatic steatosis, improved glucose homeostasis, and suppressed pro-inflammatory cytokines, independent of direct PAF exposure. These findings position gut microbiota modulation as a primary pharmacological target for NAFLD intervention.

Our study demonstrates that daily oral administration of PAF significantly reduces liver weight and ameliorates obesity-associated metabolic indices in NAFLD mice. Simultaneously, it effectively attenuates dyslipidemia and suppresses HFS-induced elevations in serum ALT and AST levels, confirming hepatoprotective effects. These findings align with previous research on paeoniflorin’s anti-steatotic properties (Jian-Ming et al., 2013; Ma et al., 2016), while extending mechanistic insights to gut-liver axis modulation. Elevated TG, TC, and LDL-C coupled with reduced HDL-C constitute established risk factors for hepatic lipid metabolism disorders. Hepatic fat accumulation originates from three primary sources: adipose tissue lipolysis, *de novo* lipogenesis (DNL), and dietary fat (Tang et al., 2023). Steatosis develops when fatty acid influx (from lipolysis or diet), DNL, and export/secretion rates exceed fatty acid oxidation capacity. *Srebp-1c*, a critical transcription factor in lipid synthesis, activates the expression of key enzymes involved in lipid metabolism, such as *Fas* and *Acc1*, thereby driving hepatic accumulation (Lally et al., 2019). Concomitantly, *Scd1* a pivotal regulator of hepatic lipid metabolism, is induced by high-fat diets and promotes steatosis through enhanced triglyceride synthesis (Zhu et al., 2019). This study found that PAF ameliorates HFS-induced NAFLD by suppressing the *SREBP1c* signaling pathway and downregulating key downstream lipogenic genes *Fas*, *Acc1*, and *Scd1*. While consistent with previous studies of PAF’s hepatoprotective effects (Ma et al., 2016; Ma et al., 2017). *Ppar*α serves as core transcriptional regulator of hepatic lipid metabolism, facilitating lipid-lowering and regulating fatty acid metabolism. Its key downstream effectors include *Cyt1*α and *Acox1* which involves in hepatic lipid metabolism. *Cpt1*α is a rate-limiting enzyme in the β-oxidation process of fatty acids, which indirectly determines the content of fatty acyl coenzyme A and affects body fat content. The *Acox1* gene encodes a key enzyme in the peroxisomal oxidation pathway. A high-fat diet can reduce the expression of *Cpt1*α and *Acox1* genes in the liver of NAFLD model group mice, indicating that high-fat diet blocks the lipid oxidative metabolism pathway of NAFLD mice. However, other study has shown that a high-fat diet does not significantly reduce the relative level of *Ppar*α gene expression (Ma et al., 2021). In this study, HFS feeding did not significantly alter hepatic *Ppar*α expression compared to the NG group. Furthermore, PAF administration also did not modulate *Acox1* transcript levels. These finding collectively indicate that PAF alleviates NAFLD associated dyslipidemia primarily through suppression of *de novo* lipid synthesis rather than via potentiation of fatty acid β-oxidation pathways.

*Srebp-2* serves as a pivotal transcription factor intricately involved in cholesterol synthesis, characterized by a long transcriptional activation domain that preferentially enhances cholesterol synthesis (Shehnaz et al., 2023). Its key target *HMGCR*, a key rate-limiting enzyme in the cholesterol biosynthesis pathway. Notably, the overexpression of *Srebp-2* in the liver resulted in a 75-fold *HMGCR* transcriptional upregulation (Horton et al., 2002). Our study found that PAF suppresses *Srebp-2* mRNA expression, thereby inhibiting *HMGCR* mRNA expression and ultimately attenuating hepatic cholesterol synthesis in NAFLD mice. *LXR*α and *SREBPs* maintain cholesterol homeostasis through antagonistic interactions. LXRα reduces cholesterol burden by facilitating reverse cholesterol transport (RCT), enhancing hepatic cholesterol-to-bile acid conversion, promoting intestinal excretion and preventing its absorption by peripheral cells via transcriptionally activates cholesterol transporters ABCA1 and ABCG1 (Zhang et al., 2012; Wang and Tontonoz, 2018). Our study revealed that PAF administration significantly upregulates LXRα expression, and significantly enhanced hepatic *ABCA1* and *ABCG1* expression in NAFLD mice, to facilitate cholesterol efflux from peripheral tissues. These findings indicate that paeoniflorin rebalances lipid metabolism by coordinately downregulating *de novo* cholesterol synthesis and upregulating RCT pathways, thereby countering HFS-induced dyslipidemia.

PAF’s glucoregulatory effects are model establishment method-dependent. While Zhang et al. (2015) observed no significant blood glucose reduction in streptozotocin-induced type 2 diabetes mellitus (T2DM) mice, Zhang et al. (2022), however, demonstrated its hepatoprotective efficacy in a high-fat diet induced T2DM mice model. Critically, our data demonstrate potent glucose metabolism modulation by PAF in high-fat diet models, evidenced by improved insulin sensitivity, reduced fasting hyperglycemia, and normalized glucose transporter expression. It should be noted that the present study did not include an insulin tolerance test (ITT), which could provide additional dynamic information on peripheral insulin sensitivity. Nevertheless, the comprehensive assessments of fasting glucose, insulin, OGTT-AUC, and HOMA-IR collectively offer robust and consistent evidence for the improvement of insulin resistance by PAF. PAF-mediated transcriptional reprograming of hepatic glucose metabolism genes underlies this metabolic benefit. Specifically, we confirmed significant downregulation of *pkm2*, *G6Pase*, and *PEPCK* mRNA in PAF-treated mice, collectively suppressing gluconeogenic flux while promoting glycolytic efficiency. These coordinated changes establish the mechanistic basis for PAF’s improvement of glucose homeostasis in HFS-fed models.

Obesity and therapeutic interventions invariably alter serum metabolite profiles, with critical implications for metabolic homeostasis. To delineate PAF’s impact on systemic metabolism, we conducted untargeted metabolomic profiling of serum samples, identifying pathway-specific alterations through integrated bioinformatic analyses. sPLS-DA analysis and volcanic maps indicated that PAF supplementation restructured the serum metabolome in NAFLD mice. KEGG enrichment analysis revealed predominant metabolite enrichment in pathways critical to energy substrate handling, including glycolipid metabolism, galactose metabolism, glycerophospholipid metabolism, glycolysis/gluconeogenesis, pyruvate metabolism, and the TCA cycle pathways. These coordinated metabolic shifts indicate PAF rectifies glycolipid metabolic disturbances, thereby conferring protection against HFS-induced NAFLD pathogenesis. Concurrently, PAF significantly modulated the metabolism of glycine, serine, and threonine. Relevant study has shown that glycine serves as a mediator between insulin resistance and diabetes, and it is a strong predictor of glucose tolerance (Merino et al., 2018; Rizo-Roca et al., 2025). These observations provide mechanistic insights into PAF’s efficacy against HFS-induced glucose intolerance and metabolic dysregulation.

Sustained hepatic lipid accumulation drives metabolic dysregulation and fatty acid oxidation-induced oxidative stress, triggering pro-inflammatory cascades. Critically, insulin resistance serves as the primary driver of exacerbated oxidative stress and inflammation in this context (Yaribeygi et al., 2020). Enhancing insulin sensitivity through pharmacological or nutritional interventions reduces oxidative stress (Polianskyte-Prause et al., 2020). Concomitantly, oxidative stress functions as a pathogenic driver in obesity, diabetes, and NAFLD progression by perpetuating mitochondrial dysfunction and pro-inflammatory signaling (Masschelin et al., 2020). This study demonstrates that PAF supplementation effectively attenuates serum oxidative stress. This finding extends previous reports of PAF’s antioxidant properties in NAFLD (Ma et al., 2017). Moreover, PAF alleviated systemic and intestinal oxidative stress in HFS-induced mice, manifested by reducing the intestinal MDA level and upregulating SOD1 and SOD2 expression. Critically, high-fat-diet-derived free fatty acids directly compromise intestinal immunity by elevating barrier-disrupting cytokines such as TNF-a, IL-1β, IL-6, and IFN-γ (Tanaka et al., 2020; Stoeva et al., 2021). Our results demonstrate that PAF significantly suppressed pro-inflammatory mediators, including TNF-α, MCP-1, IL-6, and IL-1β, across systemic circulation, hepatic, and intestinal tissues in NAFLD mice. Mechanistically, PAF reduced gut-derived LPS translocation into systemic circulation, thereby attenuating a key pathological trigger of obesity-related inflammation (Tilg et al., 2020; Chen et al., 2022). Studies indicate that a high-fat diet promotes intestinal absorption of LPS, leading to metabolic endotoxemia which induces inflammation, oxidative stress, and diabetes (Gomes et al., 2018). Increased circulating LPS levels are primarily attributed to intestinal barrier dysfunction (Ghosh et al., 2020). The Zonulin family proteins, key regulators of intestinal permeability, are implicated in various autoimmune and metabolic disorders (Fasano, 2020). Moreover, reduced expression of *ZO-1* and *Occludin* genes increases in intestinal permeability (Oliveira et al., 2019; Nascimento et al., 2021). In this study, HFS-induced NAFLD mice exhibited significantly decreased mRNA and protein expression of the intestinal tight junction proteins ZO-1, Occludin, and Claudin-1. Conversely, PAF treatment significantly inhibited the HFS-induced elevation in LPS levels. Furthermore, PAF upregulated the mRNA and protein expression of ZO-1, Occludin, and Claudin-1, thereby restoring intestinal barrier function.

The relationship between gut microbiota dysbiosis and NAFLD is established as bidirectional through the gut-liver axis, where alterations in microbial composition and function are mechanistically linked to hepatic steatosis, inflammation, and fibrogenesis (Aron-Wisnewsky et al., 2020). Animal and human studies show that the gut-liver axis influences NAFLD through processes including increased intestinal permeability, elevated circulating pathogen-associated molecular patterns (e.g., lipopolysaccharide), altered bile acid metabolism, and production of microbiota-derived metabolites (Pezzino et al., 2022; Ni et al., 2023). HFS diets reduce the gut microbial diversity in mice and commonly feature decreased Bacteroides abundance, increased Firmicutes abundance, and an elevated Firmicutes/Bacteroidetes (F/B) (An et al., 2022). Our study demonstrates that PAF supplementation significantly restored gut microbial diversity and community structure in HFS-fed mice. Specifically, PAF treatment significantly increased Bacteroidetes abundance while reducing Firmicutes abundance and F/B ratio. Notably, PAF elevated beneficial taxa including *uncultured_bacterium_f_Muribaculaceae*, *Akkermansia*, *Candidatus_Arthromitus*, and *Muribaculaceae*, which associated with improved glucose and lipid metabolism (Mu et al., 2020; Wei et al., 2023; Zabolotneva et al., 2024). Additionally, *Candidatus_Arthromitus*, segmented filamentous bacteria (SFB) originally identified in rodent intestine (Davis and Savage, 1974), and later observed as genetically distinct variants in humans (Jonsson et al., 2020), contributes to gut microbial homeostasis (Jia et al., 2024; Imdad et al., 2024). SFB enhance host immunity by stimulating Th-17 cell differentiation and IL-17 production, conferring protection against bacterial and fungal infections (Wang et al., 2019). Correlation analysis revealed significant inverse relationships between *uncultured _bacterium_f_Muribaculaceae, Akkermansia*, and *uncultured_bacterium_f_Peptococcaceae* abundance and key metabolic indicators, including serum/liver lipid parameters and oxidative stress markers. Notably, *Akkermansia* ameliorates metabolic dysfunction associated fatty liver disease via the “gut-liver axis” by enhancing lipid metabolism and improving non-alcoholic fatty liver (Rao et al., 2021). In our study, PAF treatment significantly increased *Akkermansia* abundance, which was associated with improved metabolic parameters, suggesting that PAF may exert part of its hepatoprotective effect through enriching this beneficial bacterium. Furthermore, it has been reported that *Candidatus_Saccharimonas*, *Desulfovibrio*, and *Helicobacter* are implicated as pathogenic bacteria contributors to metabolic abnormalities (Peng et al., 2021; Su et al., 2022; Yan et al., 2024). Among these, *Desulfovibrio*, which belong to the Desulfobacterota phylum, is a sulfate-reducing bacterium, endotoxin-producing bacterium, that expands significantly in mice under HFS diets. This proliferation correlates with inflammation and drives obesity-related pathologies (Yuan et al., 2021; Song et al., 2023; Zhu et al., 2024). Additionally, *Helicobacter* has been shown to promote obesity and insulin resistance (Peng et al., 2021; Dai et al., 2022). In this study, the abundance of *Desulfovibrio* and *Helicobacter* showed significant positive correlations with MDA levels, glucolipid metabolic indices, and inflammatory factors, while exhibiting negative correlations with SOD activity in NAFLD mice. Importantly, PAF treatment significantly downregulated the abundance of these harmful bacteria, which aligns with the observed amelioration of oxidative stress, metabolic dysregulation, and inflammation, thereby providing a microbial mechanism for its therapeutic effects against NAFLD. Collectively, these findings demonstrate that the PAF supplementation modulates gut microbiota by enriching the relative abundance of beneficial microorganisms while suppressing the proliferation of pathogenic genera, thereby alleviating HFS-induced metabolic dysregulation, chronic inflammation and oxidative stress in NAFLD mice.

SCFAs play a crucial role in maintaining intestinal epithelial barrier homeostasis. Acetate, propionate, and butyrate accounting for approximately 90% of microbially derived SCFAs, collectively the three predominant SCFAs (Chen et al., 2020). This study demonstrates that PAF supplementation significantly elevated intestinal levels of acetate, butyrate, isobutyrate, and total SCFAs in HFS-fed mice. Additionally, it has been shown that butyrate enhances intestinal barrier integrity by upregulating tight junction proteins *ZO-1* and *claudin-1* (Mörkl et al., 2018). Relevant studies have confirmed a significant decrease in the abundance of SCFA-producing bacteria, such as *Ruminococcaceae*, *Bacteroides*, and *Akkermansia*, in the intestines of mice on a high-fat diet. Conversely, the relative abundance of endotoxin-producing bacteria, such as *Desulfovibrio* and *Helicobacter*, has increased substantially. This dysbiotic shift drives systemic accumulation of LPS and other endotoxins (Tian et al., 2021). In this study, correlation analysis revealed significant positive correlations between *Akkermansia* abundance and intestinal concentrations of butyrate, acetate, and total SCFAs. In contrast, *Desulfovibrio* and *Helicobacter* abundance exhibited significant negative correlations with butyrate. Additionally, the concentrations of acetate and total SCFAs exhibited significant negative correlations with pro-inflammatory factors and glucose—lipid metabolic indices in NAFLD mice. Simultaneously, these SCFAs also negatively correlated with oxidative stress indices. Furthermore, microbial composition shifts coincided with functional alterations that ultimately regulate host metabolism. PICRUSt analysis revealed that PAF significantly downregulated microbial pathways for Glycolysis/Gluconeogenesis, Galactose metabolism, and Fructose and Mannose metabolism. This suppression likely reduces carbohydrate digestion and absorption in the murine gut.

To investigate whether PAF ameliorates NAFLD are mediated by the intestinal microbiota, we transplanted fecal microbiota from PAF-treated donors into HFS-fed mice. Recipients exhibited significantly enriched *Akkermansia* abundance, alongside marked reductions in *Desulfovibrio* and *Helicobacter* when compared with fecal samples from mice fed the HFS diet. Crucially, the transplantation of PAF-regulated microbiota alleviated HFS-induced metabolic disorders, systemic inflammation, and oxidative stress in NAFLD mice. Building on these findings, our study suggests promising avenues for future research. Translating PAF-modulated microbiota into clinical practice should be prioritized, including human trials to evaluate its efficacy and safety in NAFLD patients. Furthermore, the downstream mechanisms require deeper investigation, particularly the roles of SCFA receptors such as GPR43/109A and related signaling pathways like AMPK and NLRP3, to clarify how microbial metabolites mediate antioxidant and anti-inflammatory effects. Additionally, exploring synergistic strategies, such as combining PAF with specific probiotics, prebiotics, or dietary interventions, may optimize gut-liver axis modulation and improve therapeutic outcomes in metabolic liver disease. Several limitations of the present study should be acknowledged. PAF demonstrated significant therapeutic efficacy over the 10-week intervention period. However, its long-term safety and durability remain to be established. Chronic toxicity studies and extended observation periods in preclinical models are essential. Well-designed clinical trials will also be necessary to assess the translational potential of PAF for sustained NAFLD management. PAF treatment induced substantial functional remodeling of the gut microbiota. This was evidenced by the enrichment of beneficial taxa such as *Akkermansia* and the suppression of pro-inflammatory genera including Desulfovibrio and Helicobacter. However, the changes in α-diversity indices, including Shannon and Simpson, were relatively modest compared to the strong functional outcomes. This observation is consistent with a growing body of evidence suggesting that shifts in key functional species may serve as more sensitive indicators of therapeutic efficacy than overall diversity metrics alone (Dong et al., 2024). Future studies incorporating deeper metagenomic sequencing and longitudinal sampling will help clarify the relationship between diversity restoration and functional recovery.

## 5 Conclusion

In summary, this study demonstrates that PAF intervention alleviates NAFLD induced by HFS in mice by inhibiting hepatic lipogenesis and promoting cholesterol efflux, thereby normalizing serum lipid metabolites. Furthermore, PAF supplementation concurrently preserved intestinal barrier integrity by tight junction upregulation, inhibited the leakage of LPS into the bloodstream, reduced inflammation and oxidative stress levels in intestinal tissues. Critically, PAF enriched abundance of *Akkermansia*, which is a short-chain fatty acid producer that modulates lipid metabolism, while suppressing pro-inflammatory pathobionts *Desulfovibrio and Helicobacter*. This microbiota remodeling underlies PAF’s mitigation of obesity-related metabolic syndrome, revealing its therapeutic potential for metabolic disorders.

## Data Availability

The data presented in this study are publicly available. The data is deposited in National Microbiology Data Center (NMDC) with accession number NMDCX0002101 (https://nmdc.cn/resource/attachment/detail/NMDCX0002101).

## Footnotes

1
www.biocloud.net
