# Supplementary material for: Paeoniflorin protects against NAFLD through antioxidant, anti-inflammatory effects and restoration of gut microbiota homeostasis
Source: Front Microbiol. 2026 Mar 13;17:1766068. doi: 10.3389/fmicb.2026.1766068 (PMC13021628; doi:10.3389/fmicb.2026.1766068)
Supplement: Supplementary file 1 [file Supplementary_file_1.docx]

Supplementary Material

# Experimental methods

## Untargeted metabolomics analysis

Sample Extraction Buffer: (1) Weigh 100μL sample, add 500μL extraction solution containing inner target (methanol acetonitrile volume ratio =1:1, internal standard concentration 20mg/L), and swirl well for 30 seconds; (2)swirl well for 30 seconds; (3) Stand at minus 20℃ for one hour; (4) Centrifuge the sample at 12000rpm at 4℃ for 15min; (5) Carefully remove 500μL supernatant into the EP tube; (6) Drying extracts in a vacuum concentrator; (7) 160μL extraction solution (acetonitrile water volume ratio: 1:1) was added to the dried metabolites to redissolve; (8) Vortex 30 seconds, ice water bath ultrasound 10 minutes; (9) Centrifuge the sample at 12000rpm at 4℃ for 15min; (10) Carefully take out 120μL supernatant in 2mL injection bottle, and take 10μL of each sample and mix it into QC sample for machine testing.

The LMS system for metabolomics analysis consists of Waters Acquity I-Class PLUS ultra-high performance liquid chromatography in tandem with Waters Xevo G2-XS QTOF high resolution mass spectrometer. Acquity UPLC HSS T3 column (1.8um 2.1*100mm) purchased from Waters was used. Positive ion mode (POS): mobile phase A: 0.1% formic acid solution; Mobile phase B: 0.1% carboxynitrile anion mode (NEG): mobile phase A: 0.1% formic acid aqueous solution; Mobile phase B: 0.1% acetonitrile formate. The original data collected by Mass Lynx V4.2 were processed by the Progenesis QI software for peak extraction, peak alignment and other data processing operations. The identification was carried out based on the online METLIN database of Progenesis QI software, the public database and the self-built database of Baimai, and the theoretical fragment identification was also carried out. The mass number deviation of parent ion is 100ppm, and the mass number deviation of fragment ion is less than 50ppm.

# Supplementary Figures and Tables

## Supplementary Figures


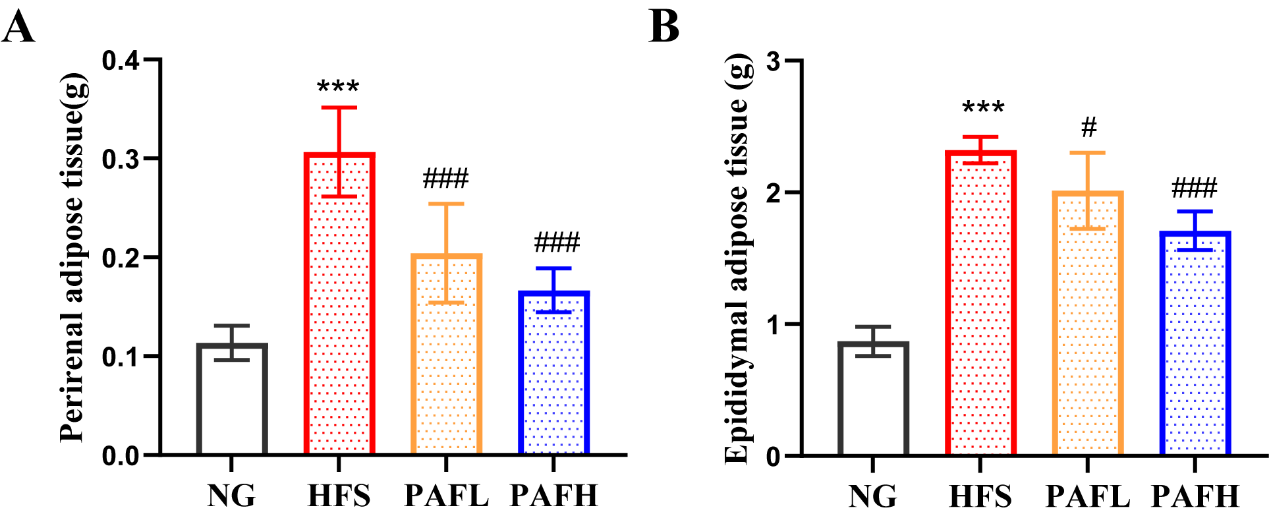


**Supplementary Figure 1.** **Effects of PAF on** **adipose tissue mass in HFS-fed mouse model.** (A) Perirenal adipose tissue mass; (B) Epididymal adipose tissue mass. NG: Normal control group; HFS: High-fat/sugar diet-induced model group; PAFL: Low-dose paeoniflorin intervention group; PAFH: High-dose paeoniflorin intervention group. Data are presented as mean ± SD. ***p < 0.001 vs. NG group; #p < 0.05, ###p < 0.001 vs. HFS group.


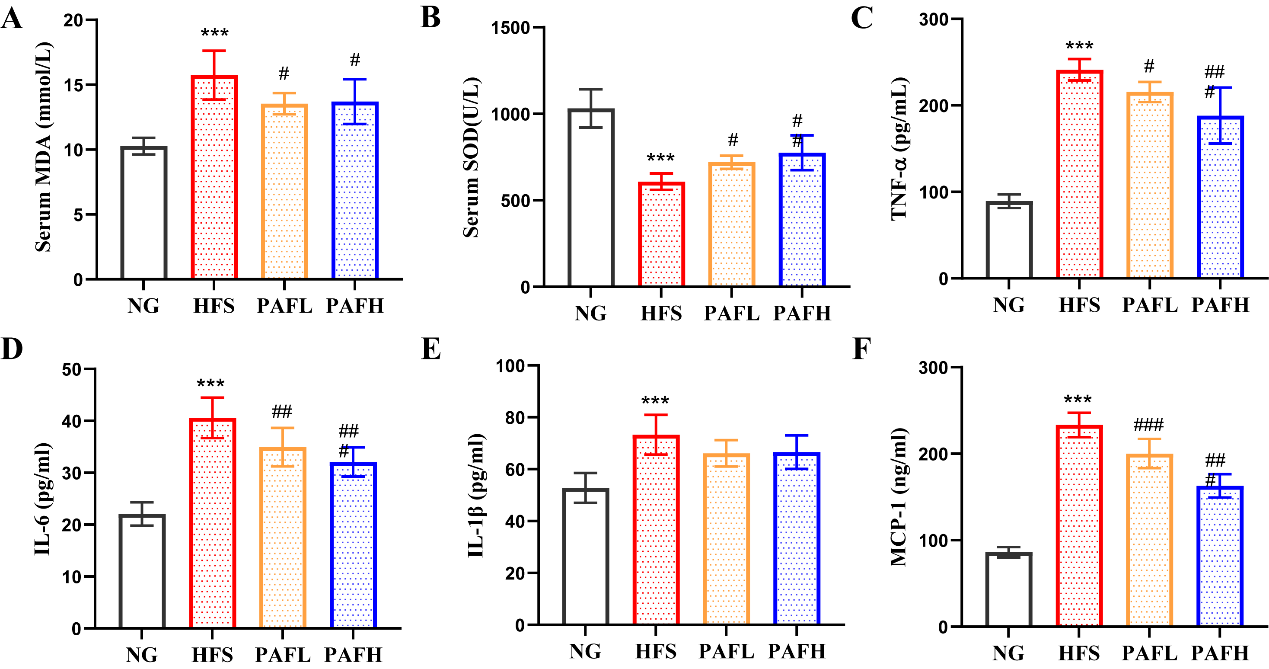


**Supplementary Figure 2.** **Effect of PAF supplementation on oxidative stress index** (A) antioxidative enzymes (B) and inflammation in serum (C-F). NG: Normal control group; HFS: High-fat/sugar diet-induced model group; PAFL: Low-dose paeoniflorin intervention group; PAFH: High-dose paeoniflorin intervention group. Data are presented as mean ± SD. **P* < 0.05, ***P* < 0.01. * HFS vs NG, ^#^PAFL or PAFH vs HFS, ^#^/**P* < 0.05, ^##^/***P* < 0.01, and ^###^/****P* < 0.001.

**
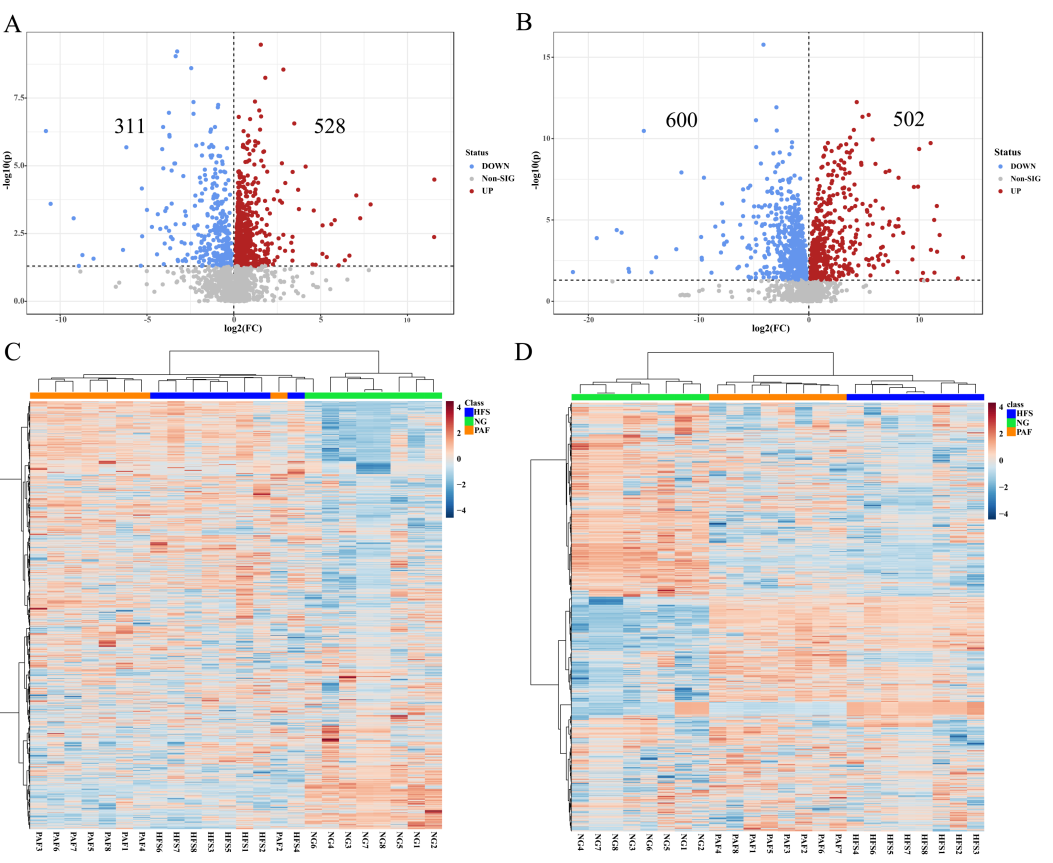
**

**Supplementary Figure 3. The serum metabolic profile altered compare NG to HFS.** (A, B) by volcano plot in positive and negative ion modes, (C, D) Differential metabolites in serum identification in different groups in positive and negative ion modes by heat maps. NG: Normal control group; HFS: High-fat/sugar diet-induced model group; PAF: High-dose paeoniflorin intervention group.


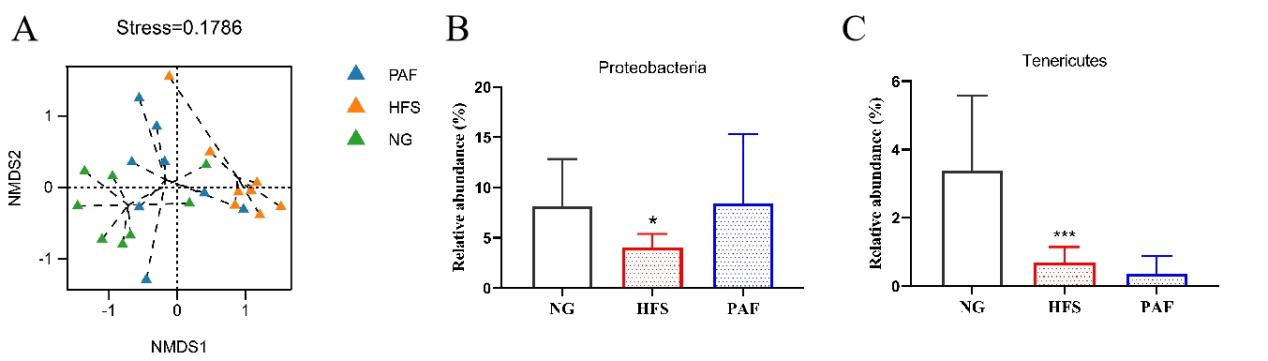


**Supplementary Figure 4. Gut microbiota analysis in PAF treated mouse.** (A) Non-metric multidimensional scaling (NMDS) result based on Bray Curtis algorithm, (B, C) the composition of the gut microbiota at phylum taxa level, **P*<0.05 and ****P* < 0.001, HFS compare with NG.


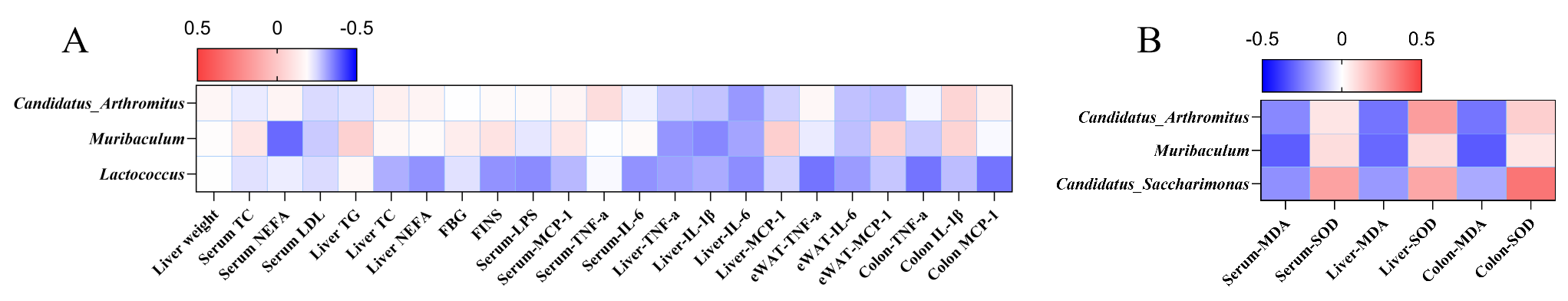


**Supplementary Figure 5. Correlation analysis between gut microbiota and biochemical index, inflammatory factors and oxidative stress indicators.** (A) Heatmap of spearman’s correlation between correlation between the gut microbiota and biochemical indexes and inflammatory factors at the genus level, (B) correlation between the gut microbiota and oxidative stress indicators at the genus level.


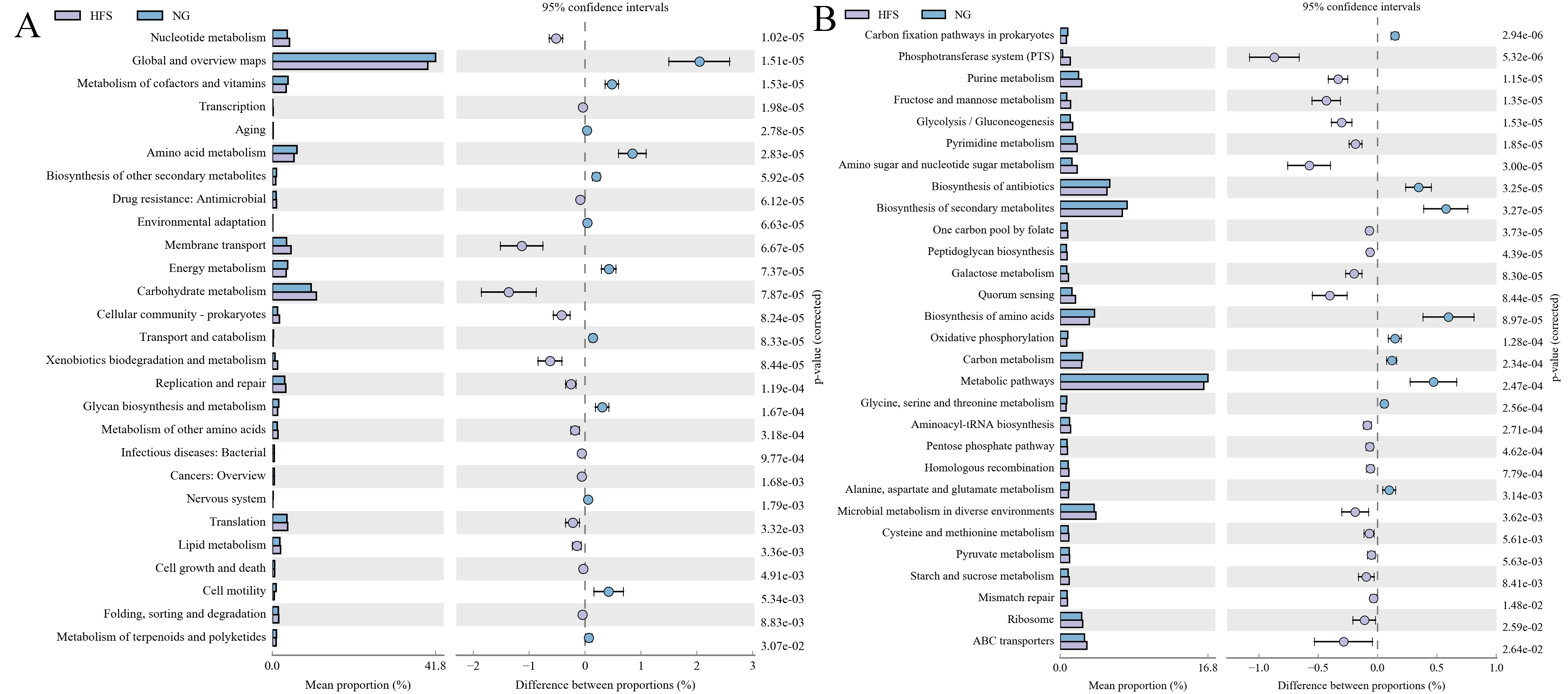


**Supplementary Figure 6. Differential KEGG pathways between the NG and HFS groups identified using PICRUSt2 prediction.** KEGG metabolic pathway difference between NG and HFS group at level 2 (A) and level 3 (B).


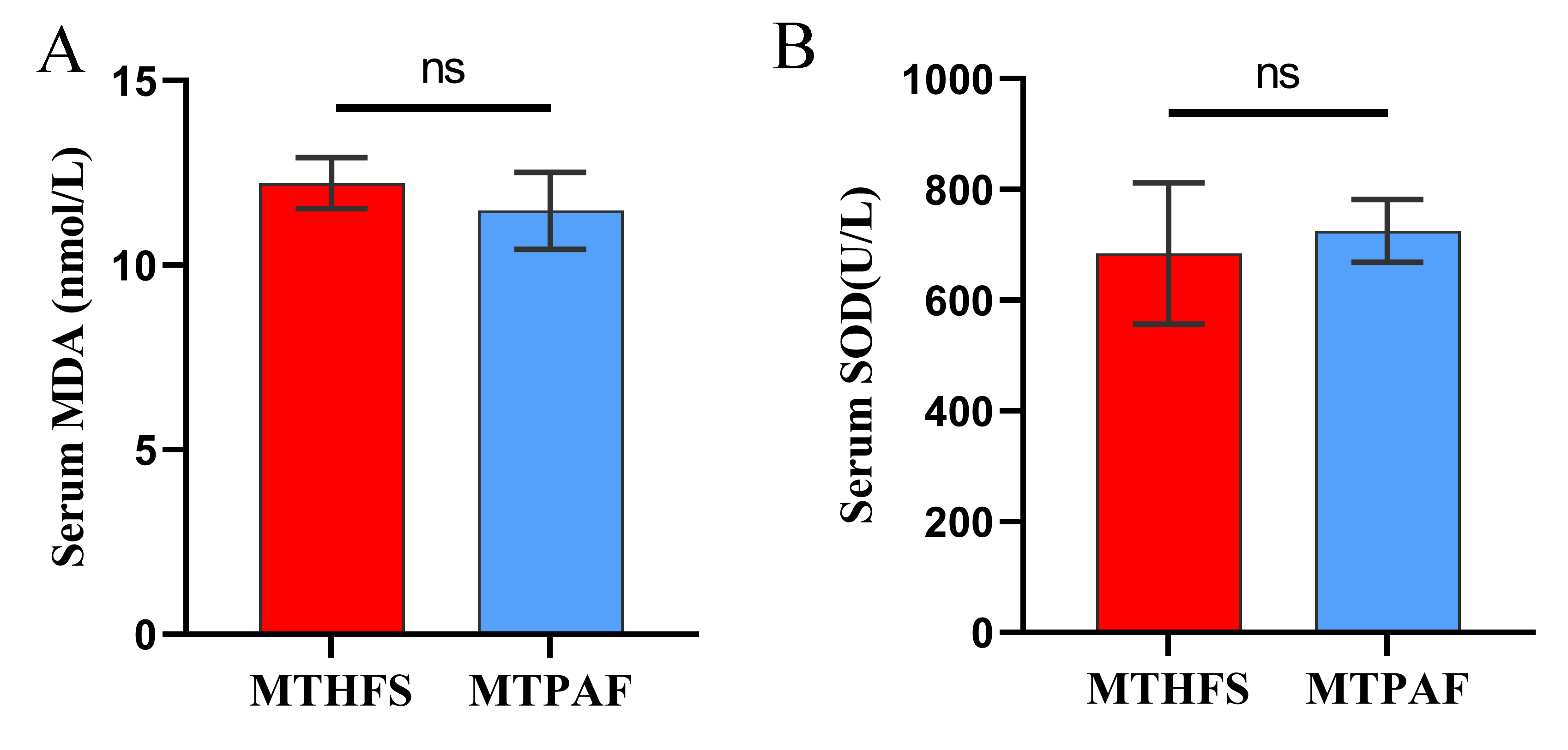


**Supplementary Figure 7. The difference between MTPAF and MFHFS in oxidative stress index.** (A) the serum MDA concentration; (B) the SOD activity in serum. ns, no significant.


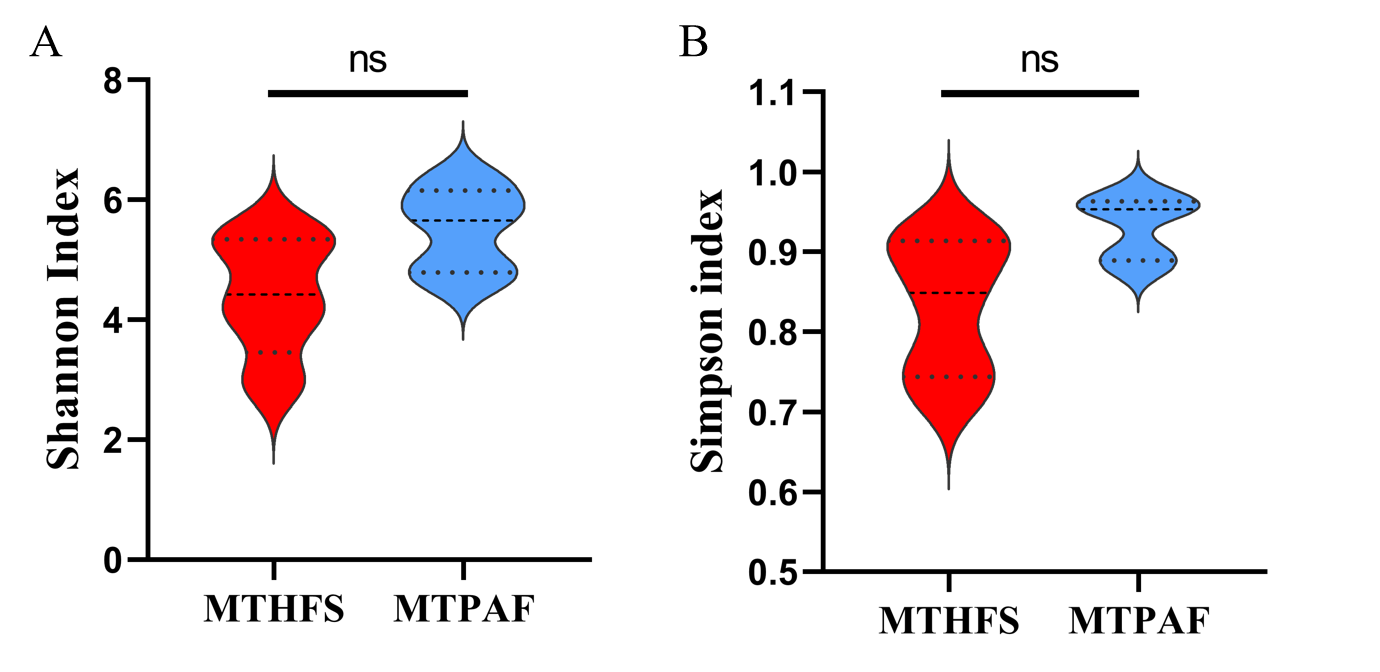


**Supplementary Figure 8. The gut microbiota α-diversity analysis in FMT experiment.** Shannon index (A) and Simpson index (B).


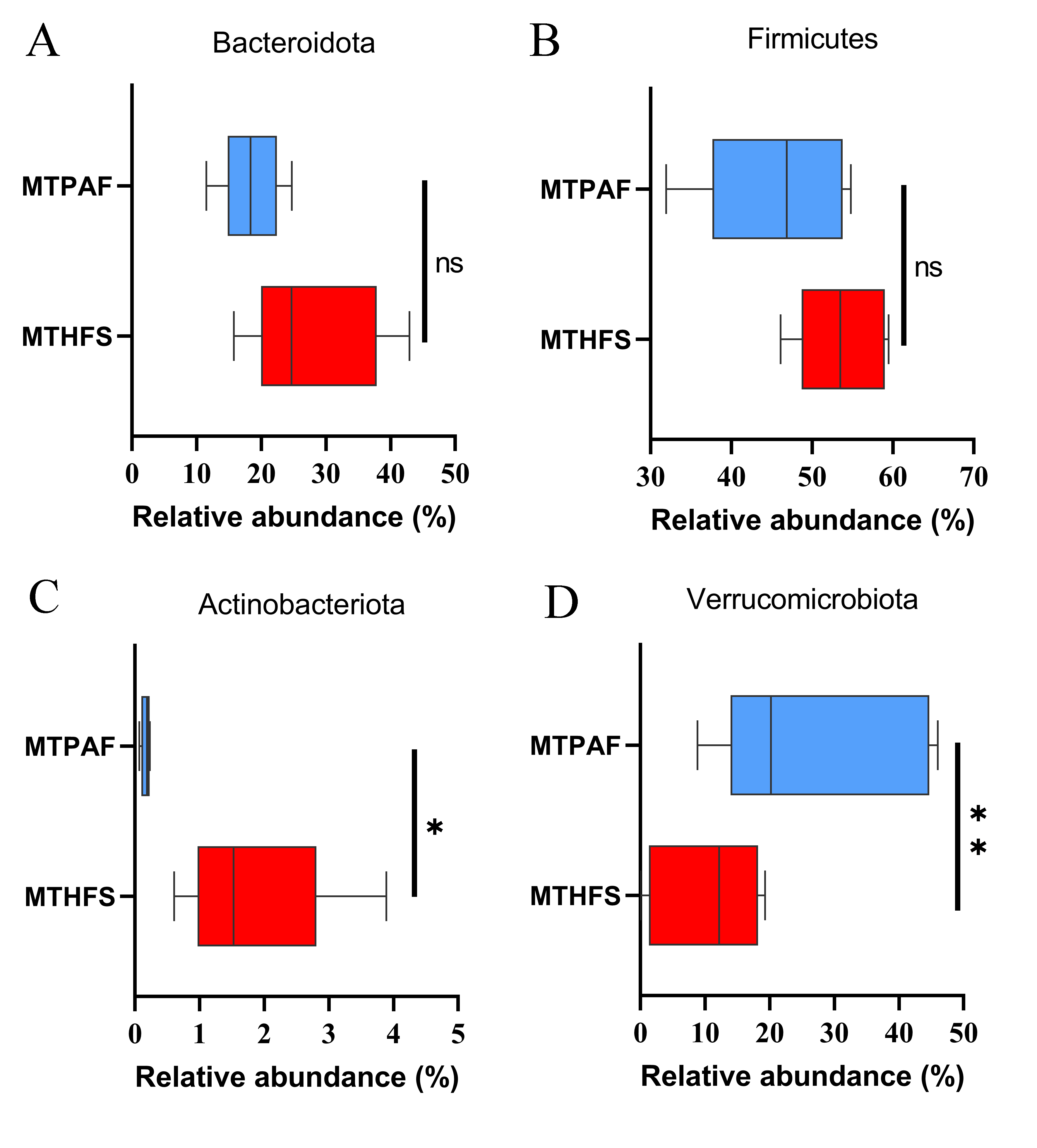


**Supplementary** **Figure 9. Changes of the composition of the gut microbiota at phylum taxa level by MTPAF**. ns, no significant; **P*<0.05 and ***P* < 0.01.

## Supplementary table

**Supplementary** **Table 1 Sequences of primers used for qPCR in this study.**

| Name | Sequence（5'-3') |
| --- | --- |
| ACC | F: TGGAGAGCCCCACACACA |
|  | R: TGACAGACTGATCGCAGAGAAAG |
| FAS | F: TGAATCAGCCCCACGCAGT |
|  | R: CCGAGTCAGTCTTGGAGGACAT |
| PPARα | F: GAGGCAGATGACCTGGAAAGT |
|  | R: TGCGTGAACTCCGTAGTGGTA |
| Acox1 | F: GTTCTCACGATGCCAATGC |
|  | R: ATGCTGGGGTTACAGGTTTG |
| SREBP-1c | F: CACTTCTGGAGACATCGCAAAC |
|  | R: GTCCTCCTGTGTACTTGCCCA |
| SCD1 | F: TCTTCCTTATCATTGCCAACACCA |
|  | R: GCGTTGAGCACCAGAGTGTATCG |
| Cpt1α | F: AGGACCCTGAGGCATCTATT |
|  | R: ATGACCTCCTGGCATTCTCC |
| Ppar γ | F: CTGGCCTCCCTGATGAATAAAG |
|  | R: AGGCTCCATAAAGTCACCAAAG |
| LXRα | F: GAGAGGCTGCAACACACATA |
|  | R: GAGGCTCACCAGCTTCATTAG |
| ABCG1 | F: GCGAAGCTGTACCTGGATTT |
|  | R: TACCTCTCAGCCCGGATTT |
| ABCA1 | F: GGGTGGTGTTCTTCCTCATTAC |
|  | R: CACATCCTCATCCTCGTCATTC |
| Pkm2 | F: GCCGCCTGGACATTGACTC |
|  | R: CCATGAGAGAAATTCAGCCGAG |
| Pgk1 | F: GAACAAGGTTAAAGCCGAGCC |
|  | R: GTGGCAGATTGACTCCTACCA |
| Gck1 | F: AGCATTCAACGCCAGGTTC |
|  | R: CGAGTCTGTCAGTTCAATACCAA |
| G6P | F: AGAGACTGTGGGCATCAATCT |
|  | R: CCGGAATCCATACGTTGATT |
| PEPCK | F: GCCGACCTCCCTTAGAAATAG |
|  | R: CGAACTTCGGAGAACAGACGTGA |
| MCP-1 | F: TCTGGACCCATTCCTTCTTG |
|  | R: TCTGGACCCATTCCTTCTTG |
| IL-6 | F: CAAGAAAGACAAAGCCAGAGTCCTT |
|  | R: CAAGAAAGACAAAGCCAGAGTCCTT |
| TNFα | F: ATAGCTCCCAGAAAAGCAAGC |
|  | R: CACCCCGAAGTTCAGTAGACA |
| IL－1β | F: CCGTGATGATGACCTGAGGAG |
|  | R: CAAGACAGGTATAGATTCTTGTC |
| ZO-1 | F: GCCGCTAAGAGCACAGCAA |
|  | R: TCCCCACTCTGAAAATGAGGA |
| Occludin | F: TTGAAAGTCCACCTCCTTACAGA |
|  | R: CCGGATAAAAAGAGTACGCTGG |
| Claudin-1 | F: GGGGACAACATCGTGACCG |
|  | R: AGGAGTCGAAGACTTTGCACT |
